# Rare-Earth-Modified Copper-Oxalate-Derived CuO Nanostructures for Rapid Methyl Orange Photodegradation Under Simulated Solar Irradiation

**DOI:** 10.3390/nano16150908

**Published:** 2026-07-24

**Authors:** Hangning Wang, Rifath Bin Hossain, Yanling Yang, Mengran Wu, Xinyu Dai, Fengxiang Qin

**Affiliations:** 1 School of Materials Science and Engineering, Nanjing University of Science and Technology, Nanjing 210094, China; whn@njust.edu.cn (H.W.); 124116011402@njust.edu.cn (M.W.); xinyudai@njust.edu.cn (X.D.); 2 School of Mechanical Engineering, Nanjing University of Science and Technology, Nanjing 210094, China; rifath.bin.hossain@njust.edu.cn; 3 Fujian Key Laboratory of Flexible Electronics, Strait Institute of Flexible Electronics (SIFE Future Technologies), Fujian Normal University, Fuzhou 350117, China; ylyang@fjnu.edu.cn

**Keywords:** rare-earth modulation, copper-oxalate-derived CuO, interfacial charge transfer, simulated solar photocatalysis

## Abstract

Efficient photocatalytic degradation of azo dyes requires coordinated control of nanostructure, surface chemical environment, and interfacial charge transport. In this work, rare-earth-modified copper-oxalate-derived CuO nanostructures (RE = Ce, Sm, Er, Tm, and Yb) were prepared through hydrothermal synthesis of a copper oxalate precursor, followed by calcination and ultrasonic-assisted RE modification. Structural, spectroscopic, optical, and electrochemical analyses show RE-associated apparent lattice perturbation, modified surface oxygen environments, stronger visible-region optical responses, higher apparent majority-carrier-density descriptors, and lower fitted interfacial charge-transfer resistance relative to pristine CuO. Among the samples, Ce-CuO exhibited the best performance, with a band gap of 1.48 eV, an apparent majority-carrier density of (1.94 ± 0.04) × 10^21^ cm^−3^, and a charge-transfer resistance of 216 Ω·cm^2^. It achieved 91.59% methyl orange (MO) decolorization within 9 min under simulated solar irradiation, corresponding to a 23.7-fold higher apparent rate constant than pristine CuO, and retained 75.8% decolorization efficiency after eight cycles. Scavenger experiments suggested that h^+^ was the principal oxidative species under the investigated conditions, while ·OH and ·O_2_^−^ also contributed to MO transformation. Overall, the results show that rare-earth modification is associated with changes in the structural, surface-chemical, optical, and interfacial electrochemical characteristics of copper-oxalate-derived CuO photocatalysts.

## 1. Introduction

The escalating problem of water pollution caused by industrial effluents containing persistent organic contaminants, particularly synthetic dyes, poses a serious threat to ecosystems and human health [1,2]. Owing to their stable aromatic structures and resistance to natural degradation, these pollutants are difficult to remove by conventional treatment processes [2]. In this context, photocatalysis has emerged as a promising remediation strategy because it can utilize solar energy to drive the degradation of refractory organic pollutants under mild conditions [3]. However, many conventional semiconductor photocatalysts still suffer from limited utilization of the solar spectrum and rapid recombination of photogenerated charge carriers. As representative examples, wide-bandgap metal oxides such as TiO_2_, ZnO, and SnO_2_ have been extensively investigated for photocatalytic applications, but their intrinsic photoresponse is largely confined to the ultraviolet (UV) region because of their relatively large band gaps [4,5,6]. Addressing this limitation requires nanostructured photocatalysts that combine efficient visible-region light absorption with improved charge-carrier utilization [3].

Among narrow-bandgap oxide photocatalysts, p-type copper oxide (CuO) has attracted significant attention because of its natural abundance, low cost, and relatively low toxicity [7]. CuO possesses a narrow band gap of approximately 1.2–1.7 eV, enabling strong absorption in the visible-light region and making it a promising candidate for solar-driven photocatalysis [8]. Previous studies on Ce-modified CuO have shown that the introduction of Ce species can alter the structural, optical, and electronic characteristics of CuO and improve its visible-light-responsive photocatalytic behavior [9]. Nevertheless, pristine CuO commonly suffers from rapid recombination of photogenerated electron–hole pairs, resulting in low quantum efficiency and limited photocatalytic activity [10]. Accordingly, strategies that regulate the local structural, surface-chemical, and interfacial electronic characteristics of CuO are of interest for improving its photocatalytic performance [11].

Coordination-precursor routes provide a practical strategy for preparing nanostructured metal oxides through controlled thermal conversion. During calcination, decomposition of the organic component and the associated release of gaseous products can promote structural reconstruction and generate aggregated oxide nanostructures. Copper oxalate is a suitable precursor for this purpose because it can be readily prepared through coordination between Cu^2+^ and oxalate species and subsequently converted into crystalline CuO through thermal treatment [12]. Such a precursor-derived route provides a simple means of regulating CuO particle morphology without assigning quantitative porosity in the absence of nitrogen adsorption–desorption measurements.

Rare-earth (RE) modification has been widely explored as a means of regulating the local structural and electronic environments of semiconductor photocatalysts [13]. RE ions such as Ce, Sm, Er, Tm, and Yb possess distinct 4f electronic configurations and coordination characteristics. Depending on their chemical state and location, RE-associated species may alter lattice parameters, surface oxygen environments, and interfacial electronic interactions [13,14,15]. Such changes have been proposed to influence carrier trapping and surface oxygen activation; however, the exact origin and function of the corresponding electronic states remain strongly dependent on the material system and the available experimental evidence. Ce-containing systems are of particular interest because the coexistence of Ce^3+^ and Ce^4+^ surface states can provide reversible electron-accepting and electron-donating characteristics. Previous studies have associated Ce mixed-valence chemistry with changes in oxide band structure, surface oxygen chemistry, and interfacial electron-transfer behavior [16,17,18]. In a proposed electron-exchange pathway, Ce^4+^ may temporarily accept an electron to form Ce^3+^, whereas subsequent electron transfer to an adsorbed acceptor can regenerate Ce^4+^. However, the occurrence and importance of this cycle remain dependent on the host oxide, Ce coordination environment, and direct experimental evidence available for the specific material system.

Previous investigations of other rare-earth-containing photocatalysts further demonstrate that the influence of RE species is strongly dependent on the host oxide and incorporation mode. Barrocas et al. reported that Ho-containing Ca_0.6_Ho_0.4_MnO_3_ films exhibited visible-light-responsive photodecolorization of Rhodamine 6G and maintained their principal crystalline features during repeated operation [19]. Gd substitution in Bi_2_WO_6_ was also reported to modify its crystal structure and visible-light-driven photocatalytic performance [20]. Together with previous Ce-modified CuO studies [9], these findings indicate that RE-associated structural and electronic changes can regulate visible-light absorption and interfacial photocatalytic processes. Nevertheless, the effects are not universal and depend on RE identity, concentration, oxidation state, host lattice, and whether the RE species are incorporated substitutionally or distributed on the oxide surface [13,19,20]. Electrochemical studies of rare-earth-containing oxide electrodes have likewise shown that lanthanide identity can influence surface redox responses and interfacial impedance, although these effects remain strongly dependent on the host oxide and measurement environment [21,22].

RE incorporation or surface modification can influence the local lattice environment through ionic-size mismatch, charge compensation, and interfacial coordination effects. Moderate lattice perturbation has been proposed to influence local charge redistribution and carrier-transport behavior in some semiconductor systems [23]. However, its effect on photogenerated charge separation requires verification using recombination-sensitive measurements [23]. Compared with conventional hydrothermal or high-temperature solid-state routes, ultrasonic-assisted treatment relies on acoustic cavitation to generate transient localized hot spots in solution. Such non-equilibrium microenvironments can promote interactions between RE-associated species and the CuO surface and may improve their dispersion [24,25].

Representative CuO-based photocatalysts have exhibited widely varying methyl orange (MO) removal or decolorization performance. Green-synthesized CuO nanoparticles were reported to achieve 96.0% and 96.4% MO removal within 24 min under UV irradiation, whereas CuO prepared by chemical precipitation achieved approximately 90% within 120 min under UV irradiation [26,27,28]. Under sunlight, green-synthesized CuO nanoparticles achieved up to 95% MO removal within 60 min, while hydrothermally prepared CuO nanorods showed approximately 22% removal within 90 min [29,30]. In a directly relevant Ce-modified system, 1 wt.% Ce-loaded CuO exhibited a reported apparent rate constant (k_obs_) of 0.075 min^−1^ for MO degradation under UV irradiation [31]. These results demonstrate that CuO-based photocatalytic performance depends strongly on catalyst composition, synthesis route, morphology, and irradiation conditions. Direct comparison among reported efficiencies and apparent rate constants nevertheless remains complicated by differences in pollutant concentration, catalyst dosage, irradiation spectrum and intensity, pH, dark-adsorption procedure, and reactor configuration [18,26,27,28,29,30,31]. Therefore, a curated literature-derived kinetic dataset was used in the present study only to provide an external reference frame for the experimentally measured k_obs_ values. This comparison was not used for mechanistic interpretation, condition-normalized performance ranking, or prediction of the prepared catalysts.

MO was selected as the model pollutant because it is a representative anionic azo dye with a distinct visible absorption band near 463 nm, enabling convenient and reproducible time-resolved monitoring of chromophore transformation by ultraviolet–visible (UV–vis) spectroscopy. MO has also been extensively used in previous CuO-based photocatalytic studies, allowing the performance of the present catalysts to be compared with a relatively broad literature base. More importantly, the use of a single model pollutant under identical reaction conditions provides a controlled basis for comparing the effects of Ce, Sm, Er, Tm, and Yb modification. This selection does not imply that MO is intrinsically superior to other anionic dyes or representative of all emerging contaminants and real wastewater matrices, which may exhibit different adsorption, optical, and transformation behaviors.

In this work, a series of RE-modified copper-oxalate-derived CuO nanostructures (RE = Ce, Sm, Er, Tm, and Yb) were prepared through hydrothermal synthesis of a copper oxalate precursor, followed by calcination and ultrasonic-assisted RE modification. The effects of RE identity on phase structure, apparent lattice perturbation, morphology, elemental distribution, surface chemical states, optical response, and electrochemical behavior were systematically examined. MO decolorization under simulated solar irradiation was used as a model reaction to evaluate photocatalytic activity, apparent kinetics, and cycling behavior, while non-purgeable organic carbon (NPOC) analysis was used to assess aqueous organic-carbon removal. Reactive-species-scavenging, optical, and electrochemical results were jointly used to discuss possible structure–property–activity relationships without treating carrier separation or a specific RE-mediated redox cycle as directly demonstrated. The experimentally measured apparent rate constants were further positioned within a curated literature-derived kinetic reference space to provide descriptive external context rather than condition-normalized ranking or mechanistic validation.

## 2. Materials and Methods

### 2.1. Materials and Reagents

All reagents employed in this study were of analytical grade and used without further purification. Copper sulfate pentahydrate (CuSO_4_·5H_2_O), oxalic acid (C_2_H_2_O_4_), cerium nitrate hexahydrate (Ce(NO_3_)_3_·6H_2_O), samarium nitrate hexahydrate (Sm(NO_3_)_3_·6H_2_O), erbium nitrate hexahydrate (Er(NO_3_)_3_·6H_2_O), thulium nitrate hexahydrate (Tm(NO_3_)_3_·6H_2_O), and ytterbium chloride hexahydrate (YbCl_3_·6H_2_O) were purchased from Sigma-Aldrich, St. Louis, MO, USA. Methyl orange (MO), used as a model organic contaminant for photocatalytic degradation tests, was obtained from Shanghai Chemical Reagent Co., Ltd., Shanghai, China. Deionized water was utilized for all solution preparation, washing, and rinsing steps throughout the experiments. All reagents were stored under controlled conditions prior to use.

### 2.2. Synthesis of Copper-Oxalate-Derived CuO

**Figure 1:**
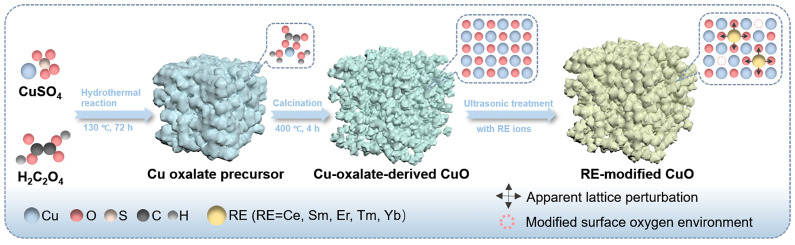
Schematic illustration of the preparation route of RE-modified copper-oxalate-derived CuO photocatalysts.

A copper oxalate precursor was synthesized using a modified hydrothermal method based on a reported procedure [12]. The complete preparation route, including hydrothermal formation of the copper oxalate precursor, calcination to obtain CuO, and subsequent ultrasonic-assisted RE modification, is schematically illustrated in Figure 1. In a typical synthesis, oxalic acid (4 mmol) and CuSO_4_·5H_2_O (4 mmol) were dissolved in 40 mL of deionized water and stirred for 30 min to obtain a homogeneous solution. The mixture was then transferred into a 50 mL Teflon-lined autoclave and maintained at 130 °C for 72 h under autogenous pressure. After natural cooling to room temperature, the resulting copper oxalate precursor was collected by filtration, thoroughly rinsed with deionized water, and dried at 60 °C for 12 h. The crystalline phase and morphology of the as-prepared precursor were examined by X-ray diffraction (XRD) and SEM, respectively, as shown in Figure S1.

The dried copper oxalate precursor was subsequently calcined at 400 °C for 4 h in air at a heating rate of 5 °C min^−1^ and then cooled naturally to room temperature. The resulting copper-oxalate-derived CuO powder was collected and used for subsequent modification and characterization.

### 2.3. Ultrasonic-Assisted RE Modification of Copper-Oxalate-Derived CuO

CuO and the corresponding rare-earth precursor were combined at a nominal Cu:RE molar ratio of 20:1, calculated on a metal-cation basis using the moles of Cu in CuO and the moles of RE in the precursor. This ratio corresponds to an RE precursor amount of 5 mol% relative to Cu, or approximately 4.76 mol% RE among the total nominal metal-cation content. This moderate nominal RE level was selected as a fixed comparative condition to enable element-to-element comparison among Ce, Sm, Er, Tm, and Yb while limiting excessive RE-rich aggregation or the formation of readily detectable separate crystalline RE-containing phases. The ratio was not established through a systematic loading-optimization study and should therefore not be interpreted as an optimized RE content. The mixture was then subjected to ultrasonication for 30 min using an ultrasonic bath (KQ2200DE, Kunshan Ultrasonic Instrument Co., Ltd., Kunshan, China; 40 kHz, 100 W) to promote the dispersion of RE-associated species and facilitate their interaction with the CuO surface. Following ultrasonication, the suspension was directly filtered without additional washing, and the collected powder was dried in a convection oven at 60 °C for 12 h, yielding RE-modified copper-oxalate-derived CuO photocatalysts. The samples are denoted as Ce-CuO, Sm-CuO, Er-CuO, Tm-CuO, and Yb-CuO according to the RE precursor used.

### 2.4. Material Characterization

The crystalline phases of all samples were analyzed by XRD with a D8 Advance (Bruker AXS GmbH, Karlsruhe, Germany) diffractometer employing Cu Kα radiation. For the selected diffraction reflections, the interplanar spacing was calculated using Bragg’s law:

(1) λ=2dsinθ

where λ is the Cu Kα wavelength, d is the interplanar spacing, and θ is the Bragg angle.

The relative interplanar-spacing change of each RE-modified sample with respect to pristine CuO was calculated according to:

(2) $$\delta =\frac{d-d_{0}}{d_{0}}\times 100\%$$

where $d_{0}$ and d are the interplanar spacings of pristine CuO and the corresponding RE-modified CuO sample, respectively. Under this sign convention, a positive δ indicates a relative expansion of the interplanar spacing, whereas a negative δ indicates a relative contraction.

Peak positions and full widths at half maximum (FWHM) were obtained by pseudo-Voigt profile fitting with a local linear background using JADE 6.0 (Materials Data, Inc., Livermore, CA, USA). The apparent coherent-domain size was first estimated using the Scherrer equation [32]:

(3) $$D_{S}=\frac{K\lambda }{\beta cos\theta }$$

where $D_{S}$ is the apparent Scherrer crystallite size, K is the shape factor taken as 0.90, λ is the Cu Kα wavelength (0.15406 nm), and β is the fitted FWHM expressed in radians. The mean $D_{S}$ and its standard deviation were calculated from the selected $\left(-111\right)$, $\left(111\right)$, $\left(-202\right)$, $\left(202\right)$, and $\left(-113\right)$ reflections. The Williamson–Hall uniform-deformation model was additionally applied according to [33]:

(4) $$\beta cos\theta =\frac{K\lambda }{D_{WH}}+4\varepsilon_{WH}sin\theta$$

where $D_{WH}$ is the apparent Williamson–Hall crystallite size and $\varepsilon_{WH}$ is the signed apparent microstrain. Linear fitting of βcosθ against 4sinθ yielded $\varepsilon_{WH}$ from the slope and $D_{WH}$ from the intercept. Because instrumental broadening was not independently corrected and the Williamson–Hall linearity was limited for several samples, the resulting crystallite-size and microstrain values are interpreted as comparative apparent estimates.

Surface morphology and microstructural features were examined by field-emission SEM using a Quanta FEG250 (FEI Company, Hillsboro, OR, USA) instrument equipped with energy-dispersive spectroscopy (EDS) for elemental analysis. EDS elemental mapping was additionally performed for the Ce-CuO sample to examine the spatial distributions of Cu, O, and Ce within selected particle aggregates. The Ce-CuO catalyst recovered after the eighth photocatalytic cycle was further characterized by XRD, SEM, and EDS to evaluate its post-reaction crystalline phase, morphology, and local semi-quantitative elemental composition.

Surface chemical states were analyzed by X-ray photoelectron spectroscopy (XPS) with a ESCALAB 250Xi (Thermo Fisher Scientific, Waltham, MA, USA) using monochromatic Al Kα excitation (hν=1486.6 eV). All XPS binding energies were charge-corrected by referencing the adventitious-carbon C 1s C–C/C–H component to 284.8 eV, and peak deconvolution was subsequently performed using XPSPEAK 4.0 (Raymund W. M. Kwok, Department of Chemistry, The Chinese University of Hong Kong, Shatin, Hong Kong, China).

Optical absorption characteristics were evaluated using UV–vis diffuse reflectance spectroscopy (UV–vis DRS) on a UV-3600i Plus (Shimadzu Corporation, Kyoto, Japan). The apparent optical band gap was estimated using the direct-allowed Tauc relation [34]:

(5) $${\left(\alpha h\nu \right)}^{2}=B\left(h\nu -E_{g}\right)$$

where α denotes the absorption-related function derived from the diffuse-reflectance data, hν is the photon energy, B is a proportionality constant, and $E_{g}$ is the apparent optical band gap. The selected linear absorption-edge region was extrapolated to (αhν)^2^ = 0 to obtain $E_{g}$. These values are interpreted as internally consistent apparent estimates because they depend on the assumed transition type and the selected fitting region.

Electrochemical measurements, including Mott–Schottky and electrochemical impedance spectroscopy (EIS), were performed using a CS350H electrochemical workstation (Wuhan Corrtest Instruments Co., Ltd., Wuhan, China). The catalyst powders were deposited on a glassy-carbon working electrode with a circular exposed diameter of 2.8 mm, corresponding to a geometric electrode area ($A_{e}$) of $0.0616\;cm^{2}$. The same exposed geometric area was used for all samples. For the p-type CuO electrodes, the space-charge capacitance was analyzed using the Mott–Schottky relation [35,36,37]:

(6) $$\frac{1}{C^{2}}=-\frac{2}{e\varepsilon_{r}\varepsilon_{0}N_{A}A_{e}^{2}}\left(E-E_{fb}-\frac{k_{B}T}{e}\right)$$

where C is the space-charge capacitance, e is the elementary charge, $\varepsilon_{r}$ is the relative dielectric constant, $\varepsilon_{0}$ is the vacuum permittivity, $N_{A}$ is the apparent acceptor density, E is the applied potential, $E_{fb}$ is the apparent flat-band potential, $k_{B}$ is the Boltzmann constant, and T is the absolute temperature. The apparent acceptor density was calculated from the magnitude of the negative slope (m) of the selected linear region according to:

(7) $$N_{A}=\frac{2}{e\varepsilon_{r}\varepsilon_{0}A_{e}^{2}\left|m\right|}$$

A relative dielectric constant of $\varepsilon_{r}=12$ was adopted uniformly for pristine CuO and all RE-modified CuO samples. Published CuO studies have employed relative dielectric constants of 10.26 and 18.1 in Mott–Schottky calculations [38,39]. The value adopted here was retained as a fixed literature-informed approximation and applied identically to all samples. Because sample-specific dielectric constants were not independently measured, the calculated $N_{A}$ values are interpreted as comparative Mott–Schottky-derived apparent majority-carrier-density descriptors rather than exact intrinsic bulk concentrations.

To assess the sensitivity of the calculated apparent majority-carrier-density descriptor to the selection of the Mott–Schottky linear region, three adjacent negative-slope regions were fitted for each original Mott–Schottky curve. The carrier density was calculated separately from each fitting region using the same electrode area, relative dielectric constant, and calculation procedure. The resulting mean, fitting-range standard deviation, relative standard deviation, and coefficient-of-determination range were used to evaluate the sensitivity of the calculated descriptor to linear-region selection. This analysis represents fitting-range uncertainty from a single electrochemical measurement rather than the standard deviation of independent Mott–Schottky measurements. The detailed numerical results are presented and discussed in Section 3.3. This fitting-range sensitivity assessment follows published cautions that Mott–Schottky outputs can depend strongly on the selected frequency and linear region [36,37].

The apparent flat-band potential was calculated from the extrapolated x-axis intercept ($E_{int}$) of the selected negative-slope Mott–Schottky region according to:

(8) $$E_{fb}=E_{int}-\frac{k_{B}T}{e}$$

where $\frac{k_{B}T}{e}=0.0257\;V$ at 298 K. For the p-type samples, the apparent Fermi-level position was approximated as:

(9) $$E_{F}\approx E_{fb}$$

For internally consistent comparison, the apparent valence-band and conduction-band positions were estimated using:

(10) $$E_{VB}\approx E_{fb}+0.10\;V\;$$

(11) $$E_{CB}=E_{VB}-E_{g}$$

All electrochemical potentials are reported versus Ag/AgCl. The resulting $E_{F}$, $E_{VB}$, and $E_{CB}$ values are treated as comparative apparent estimates rather than direct absolute energy-level measurements obtained by ultraviolet photoelectron spectroscopy, valence-band XPS, Kelvin-probe, or work-function analysis. The 0.10 V offset in Equation (10) is an empirical approximation used only for internal comparison among the p-type samples. The EIS Nyquist spectra were fitted using the same equivalent-circuit model in ZView 2 (Scribner Associates, Inc., Southern Pines, NC, USA) to obtain comparative charge-transfer resistance (R_ct_) values.

### 2.5. Photocatalytic Performance Evaluation

Photocatalytic performance was assessed using MO as a representative anionic azo-dye model pollutant in aqueous solution. A total of 10 mg of photocatalyst was dispersed in 10 mL of MO solution ($10\;mg\;L^{-1}$), corresponding to a fixed catalyst concentration of $1.0\;g\;L^{-1}$, within a photocatalytic reactor (BL-GHX-V, Shanghai Bilang Instrument Manufacturing Co., Ltd., Shanghai, China) equipped with a 1000 W xenon lamp to provide simulated solar irradiation. The same catalyst loading, solution volume, stirring conditions, and reactor geometry were maintained for all samples to enable comparative evaluation. This loading was used as a fixed experimental condition and was not established as an optimized catalyst dosage.

Prior to irradiation, the catalyst/MO suspension was magnetically stirred in the dark for 5 min to establish near-equilibrium adsorption, as verified by independent dark-adsorption experiments. The MO concentration measured after this dark-treatment period was defined as $C_{0}$ for the subsequent photocatalytic experiment. Independent dark-adsorption tests were conducted under otherwise identical conditions for pristine CuO, Ce-CuO, Sm-CuO, Er-CuO, Tm-CuO, and Yb-CuO. The suspensions were continuously stirred without irradiation, and aliquots were collected at 0, 5, 10, 20, and 30 min. After centrifugation, the residual MO concentration was determined from the absorbance at approximately 463 nm. The normalized concentration during the independent dark-adsorption experiment was calculated using:

(12) $$\frac{C_{t}}{C_{initial}}=\frac{A_{t}}{A_{initial}}$$

where $C_{initial}$ and $A_{initial}$ are the MO concentration and absorbance before contact with the catalyst, respectively, and $C_{t}$ and $A_{t}$ are the corresponding values after dark adsorption for time t.

During photocatalytic irradiation, the total irradiation period was 9 min for Ce-CuO and 12 min for pristine CuO, Sm-CuO, Er-CuO, Tm-CuO, and Yb-CuO. Aliquots of the suspension were withdrawn at predetermined time intervals, centrifuged at 4000 rpm for 4 min, and analyzed. The residual MO concentration was monitored using a UV–vis spectrometer (T6, Beijing General Instrument Co., Ltd., Beijing, China) by measuring the absorbance at approximately 463 nm. The MO decolorization efficiency (η) was calculated according to:

(13) $$\eta =\frac{A_{0}-A_{t}}{A_{0}}\times 100\%=\frac{C_{0}-C_{t}}{C_{0}}\times 100\%$$

where $A_{0}$ and $C_{0}$ are the absorbance and MO concentration after the 5 min dark-adsorption period, respectively, and $A_{t}$ and $C_{t}$ are the corresponding values after irradiation for time t. The concentration ratios were obtained from the absorbance ratios within the linear Beer–Lambert range.

The photocatalytic kinetic data were analyzed using the apparent pseudo-first-order relation:

(14) $$ln\left(\frac{C_{0}}{C_{t}}\right)=k_{obs}t$$

where k_obs_ is the condition-specific apparent pseudo-first-order rate constant. The k_obs_ value and the corresponding coefficient of determination, R^2^, were obtained from the linear regression of ln(C_0_/C_t_) against irradiation time. The photocatalytic decolorization experiments for pristine CuO and all RE-modified CuO samples were independently performed three times using separately prepared catalyst/MO suspensions under otherwise identical conditions. The apparent k_obs_ value was determined separately for each independent experiment, and the replicate profiles and statistical parameters were subsequently used for the reproducibility analysis presented in Section 3.2.

To evaluate aqueous organic-carbon removal, non-purgeable organic carbon (NPOC) analysis was performed using a total organic carbon analyzer operated in NPOC mode (TOC-L CPH, Shimadzu Corporation, Kyoto, Japan). The initial MO solution and the supernatant obtained after 9 min of photocatalytic treatment with Ce-CuO under simulated solar irradiation were analyzed. Before measurement, the treated suspension was centrifuged to remove suspended catalyst particles. The apparent aqueous NPOC reduction was calculated according to:

(15) $$\eta_{NPOC}=\frac{C_{NPOC,0}-C_{NPOC,t}}{C_{NPOC,0}}\times 100\%$$

where $C_{NPOC,0}$ and $C_{NPOC,t}$ are the NPOC concentrations of the initial MO solution and the Ce-CuO-treated supernatant, respectively. This quantity is reported as an apparent aqueous organic-carbon reduction and is not interpreted as proof of complete mineralization or detoxification.

For the cycling experiments, each photocatalytic run was conducted for 9 min for Ce-CuO and 12 min for pristine CuO, Sm-CuO, Er-CuO, Tm-CuO, and Yb-CuO. These sample-specific irradiation durations were kept unchanged throughout all eight cycles. After each run, the catalyst was recovered by centrifugation, washed three times with deionized water, and reused under otherwise identical reaction conditions. After the eighth cycle, the recovered Ce-CuO catalyst was collected for post-cycling XRD, SEM, and EDS characterization.

Additional temperature-dependent photocatalytic experiments were conducted at selected temperatures within the range of 303–333 K under otherwise identical conditions. The temperature-specific apparent pseudo-first-order rate constant ($k_{T}$) was obtained using Equation (14). Its temperature dependence was described by the Arrhenius relation:

(16) $$k_{T}=Aexp\left(-\frac{E_{a}}{RT}\right)$$

which was linearized for plotting as:

(17) $$-lnk_{T}=\frac{E_{a}}{R}\left(\frac{1}{T}\right)-lnA$$

where A is the pre-exponential factor, $E_{a}$ is the apparent activation energy, R is the gas constant ($8.314\;J\;mol^{-1}\;K^{-1}$), and T is the absolute temperature. In the plot of $-lnk_{T}$ against 1/T, the slope corresponds to $E_{a}/R$.

To elucidate the photocatalytic degradation mechanism, reactive-species-scavenging experiments were conducted for Ce-CuO, Sm-CuO, and Er-CuO by introducing 1 mM of specific scavengers into the reaction system: benzoquinone (BQ), silver nitrate (AgNO_3_), ammonium oxalate (AO), and isopropyl alcohol (IPA) as scavengers for ·O_2_^−^, e^−^, h^+^, and ·OH, respectively. The irradiation duration was 9 min for Ce-CuO and 12 min for Sm-CuO and Er-CuO, consistent with the corresponding catalyst-specific decolorization tests.

### 2.6. Literature-Derived Kinetic Benchmarking

The source dataset was obtained from the publicly available Supplementary Materials of Jiang et al. [40]. A curated literature-derived photocatalytic degradation dataset was used to provide an external kinetic reference for the experimentally measured k_obs_ values. After removal of exact duplicate entries, 439 records from 54 literature references were retained. The reported rate constants were standardized to min^−1^ and transformed as log_10_(k) for distribution-based comparison. Reference-grouped regression evaluation was additionally conducted to assess whether the heterogeneous literature descriptors supported generalizable prediction without information leakage among records from the same source. Because the resulting predictive robustness was weak, the model outputs were not used to predict the performance of the prepared catalysts or to support mechanistic conclusions. Supporting details on dataset construction, descriptive benchmarking, reference-grouped model evaluation, and sample-level contextualization are presented and discussed in Section 3.4 and the Supplementary Materials. Computational analyses were performed using Python 3.10.20 with NumPy 1.26.4, pandas 2.2.2, SciPy 1.13.1, scikit-learn 1.4.2, statsmodels 0.14.2, Matplotlib 3.8.4, Joblib 1.5.1, XGBoost 2.0.3, LightGBM 4.3.0, CatBoost 1.2.5, Optuna 3.6.1, and SHAP 0.45.1.

## 3. Results and Discussion

### 3.1. Structural, Morphological, and Surface Chemical Characterization

**Figure 2:**
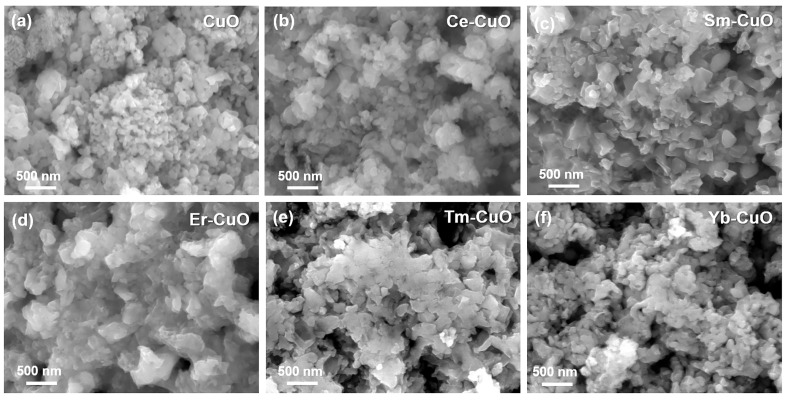
SEM images of pristine and RE-modified copper-oxalate-derived CuO photocatalysts: (**a**) CuO, (**b**) Ce-CuO, (**c**) Sm-CuO, (**d**) Er-CuO, (**e**) Tm-CuO, and (**f**) Yb-CuO.

The XRD pattern and SEM image of the as-prepared copper oxalate precursor are provided in Figure S1. The diffraction pattern confirms that a crystalline precursor was obtained before calcination, while the SEM image shows an aggregated particulate morphology. These precursor results support the copper-oxalate-derived synthesis route used to obtain the final CuO photocatalysts. Figure 2 shows the morphologies of pristine CuO and the RE-modified CuO samples. The copper-oxalate-derived CuO consists of aggregated nanoscale particles with an irregular and relatively open surface morphology. Interparticle voids can be visually observed among the aggregated particles; however, these SEM observations are not used to assign a quantitative pore structure or pore-size distribution. Following modification with Ce, Sm, Er, Tm, and Yb species, the overall aggregated morphology is retained, although differences in particle aggregation, surface roughness, and local particle features are observed among the samples. For example, Sm-CuO and Tm-CuO exhibit discernible variations in local aggregation and surface texture compared with pristine CuO. These observations indicate that RE modification influences the particle morphology without causing obvious large-scale structural collapse.

The corresponding EDS spectra and local semi-quantitative elemental compositions are presented in Figure S2 and Table S1, respectively. Pristine CuO shows a Cu:O atomic ratio of 52.2:47.8, which is approximately consistent with CuO within the semi-quantitative limitation of EDS analysis. In the RE-modified samples, the apparent Cu content decreases while the O content increases, accompanied by detectable but low apparent RE concentrations. For example, Ce-CuO contains 37.8 at.% Cu, 61.0 at.% O, and 1.2 at.% Ce. Although these values should be interpreted cautiously because of the local semi-quantitative nature of EDS analysis, the compositional variations support the introduction of low-level RE-associated species into the CuO system. Despite these variations, the overall aggregated particle morphology remains recognizable after RE modification, indicating that the treatment did not cause obvious collapse or large-scale reconstruction of the CuO aggregates. To further examine the spatial distribution of the RE species, EDS elemental mapping was performed for the as-prepared Ce-CuO sample, which exhibited the highest photocatalytic activity among the investigated catalysts. As shown in Figure S3, the Ce signal is detectable across the analyzed region, while the Cu and O signals are distributed throughout the same particle-aggregate area. No obvious micron-scale Ce-rich segregated domains are observed at the mapping resolution, supporting a broad spatial distribution of low-level Ce-associated species in the examined region. However, because EDS mapping does not provide atomic-scale structural information, these results cannot distinguish lattice-incorporated Ce from highly dispersed surface species or amorphous Ce-containing domains. The mapping results are therefore interpreted as evidence of Ce introduction and spatial distribution rather than direct proof of lattice substitution.

**Figure 3:**
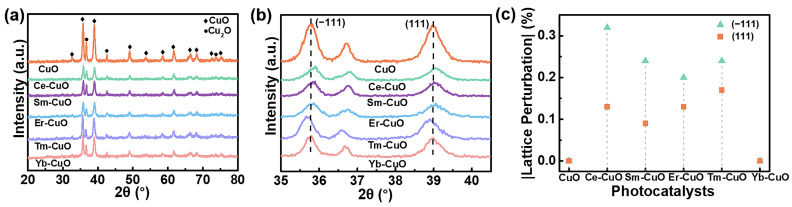
Structural characterization of pristine and RE-modified copper-oxalate-derived CuO photocatalysts: (**a**) full-range XRD patterns, (**b**) enlarged views of the (−111) and (111) reflections, with the vertical dashed lines indicating the corresponding peak positions of pristine CuO, and (**c**) apparent lattice-perturbation magnitudes calculated from the fitted diffraction peak positions.

As shown in Figure 3a, all photocatalysts exhibit two principal diffraction peaks at 2θ ≈ 35.78° and 38.99°, corresponding to the (−111) and (111) planes of monoclinic CuO (JCPDS No. 01-089-5897), confirming the formation of crystalline CuO from the copper oxalate precursor. A weak diffraction peak at 36.72° is observed for pristine CuO and is assigned to a trace Cu_2_O impurity phase (JCPDS No. 00-005-0667), which may originate from partial reduction or incomplete oxidation during thermal treatment [41,42]. No additional diffraction peaks attributable to separate crystalline rare-earth oxides or Cu–RE–O ternary compounds were detected in the modified samples. Therefore, no distinct crystalline ternary phase was identified within the XRD detection limit. Nevertheless, the absence of additional peaks does not exclude highly dispersed RE-associated surface species, amorphous RE-containing domains, or minor phases below the XRD detection limit. Accordingly, the prepared samples are described as RE-modified CuO materials containing low-level RE-associated species rather than as crystalline ternary Cu–RE–O compounds. Together with the low apparent RE contents detected by EDS, these results are consistent with the intended low-loading modification regime established using the nominal Cu:RE ratio of 20:1. Because no RE-loading series was investigated, this ratio is regarded as a fixed comparative synthesis condition rather than an optimized composition.

The slight peak shifts observed after RE modification indicate apparent lattice perturbation rather than unambiguous lattice substitution. To visualize these subtle displacements, enlarged views of the (−111) and (111) reflections are provided in Figure 3b. The vertical dashed lines indicate the corresponding peak positions of pristine CuO, whereas the arrows show the directions of displacement after RE modification. Relative to pristine CuO, Ce-CuO, Sm-CuO, and Er-CuO exhibit slight positive shifts of the (−111) reflection, while Tm-CuO shows a small shift toward lower angles. The (−111) peak of Yb-CuO remains close to that of pristine CuO but occurs at a lower angle than the corresponding peaks of Ce-CuO, Sm-CuO, and Er-CuO. These RE-dependent and non-uniform peak shifts may reflect the combined effects of ionic-size mismatch, local lattice relaxation, and defect-related charge compensation [43,44,45].

As summarized in Table S2 and illustrated in Figure 3c, RE-modified samples exhibit measurable deviations in interplanar spacing relative to pristine CuO (d_−111_ = 0.2506 nm, d_111_ = 0.2307 nm), indicating RE-associated apparent lattice perturbation. The perturbation magnitude varies with RE species and crystallographic plane. Ce-CuO exhibits the largest absolute perturbation on the (−111) plane (≈0.32%), whereas Tm-CuO shows the largest absolute perturbation on the (111) plane (≈0.17%). In contrast, Yb-CuO shows negligible perturbation on both planes. This non-monotonic behavior likely arises from the interplay of ionic radius mismatch, lattice relaxation, and defect compensation associated with different RE species. Comparable RE-dependent changes in crystal structure and photocatalytic behavior have been reported for Ce-modified CuO [9], Gd-substituted Bi_2_WO_6_ [20], and Ho-containing Ca_0_._6_Ho_0_._4_MnO_3_ visible-light photocatalysts [19]. These previous studies support the broader principle that RE incorporation can influence the local structural and electronic environment of oxide photocatalysts. However, unlike compositionally defined substitutional systems, the slight XRD shifts and EDS mapping obtained in the present work do not independently prove that the introduced RE species substitute for Cu within the CuO lattice.

The apparent crystallite size and peak-broadening-related microstrain were evaluated using five relatively isolated monoclinic CuO reflections assigned to the (−111), (111), (−202), (202), and (−113) planes. The weak Cu_2_O-related impurity reflection was excluded from the analysis. To maintain consistency with the diffraction-peak-position analysis, the previously determined 2θ positions of the (−111) and (111) reflections were retained. The selected diffraction peaks were fitted using pseudo-Voigt profiles with local linear backgrounds, and the fitted full widths at half maximum were converted from degrees to radians before calculation.

XRD peak-broadening analysis was further conducted to estimate the apparent coherent-domain size and microstrain of the samples. As shown in Figure S4a and summarized in Table S3, the mean Scherrer crystallite sizes were 18.66 ± 4.00 nm for pristine CuO, 18.82 ± 6.15 nm for Ce-CuO, 15.07 ± 3.19 nm for Sm-CuO, 14.39 ± 2.01 nm for Er-CuO, 16.73 ± 3.55 nm for Tm-CuO, and 21.85 ± 3.84 nm for Yb-CuO. The corresponding Williamson–Hall estimates were 26.38, 11.99, 10.25, 10.29, 14.62, and 17.58 nm, respectively. Thus, both approaches indicated coherent-domain dimensions within the nanometer range, although quantitative differences were observed because the Scherrer equation attributes peak broadening primarily to finite domain size, whereas the Williamson–Hall treatment additionally includes a strain-related contribution. The signed apparent Williamson–Hall microstrain values are compared in Figure S4b, and the corresponding Williamson–Hall plots are provided in Figure S5. Pristine CuO exhibited a positive fitted microstrain value of +1.500 × 10^−3^, whereas the RE-modified samples exhibited negative fitted values ranging from −0.526 × 10^−3^ to −2.405 × 10^−3^. However, the Williamson–Hall R^2^ values ranged only from 0.009 to 0.424, indicating limited linearity. These microstrain values are therefore treated as comparative indicators of peak-broadening behavior rather than precise intrinsic lattice-strain values, and the negative slopes are not considered definitive evidence of compressive strain. Neither the apparent crystallite-size trend nor the apparent microstrain trend followed the photocatalytic-activity order monotonically, indicating that these structural descriptors alone do not determine the observed photocatalytic performance.

**Figure 4:**
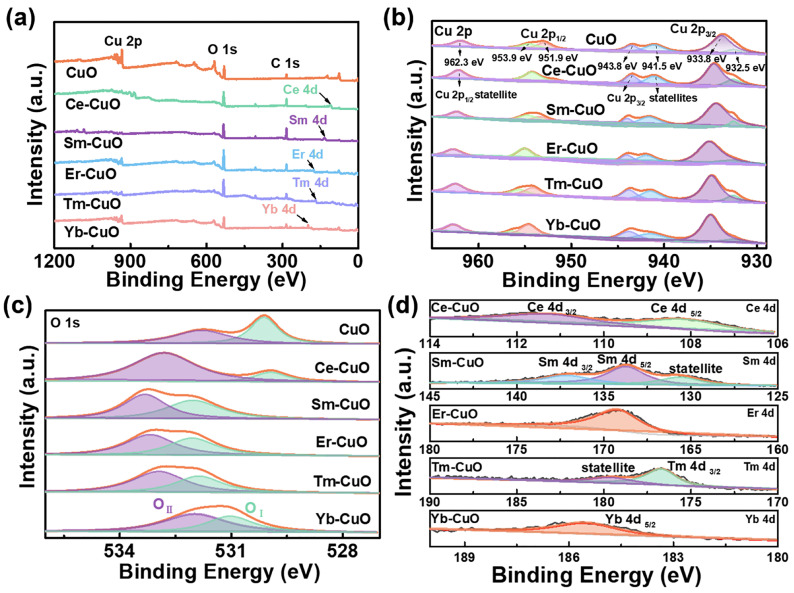
XPS characterization of pristine and RE-modified copper-oxalate-derived CuO photocatalysts: (**a**) survey spectra and high-resolution spectra of (**b**) Cu 2p, (**c**) O 1s, and (**d**) RE 4d core levels. In panel (**a**), black arrows indicate the RE 4d features. In panels (**b**–**d**), the colored filled curves represent the deconvoluted spectral components, the orange curves represent the overall fitted envelopes, and the dashed black arrows in panel (**b**) indicate the labeled Cu 2p peak and satellite positions.

The observed apparent lattice perturbation indicates that RE modification alters the local structural environment of CuO. However, its influence on photogenerated carrier separation cannot be directly established from XRD and is considered only as a possible contributing factor [23]. Figure 4 presents XPS analyses of pristine and RE-modified CuO, revealing insights into chemical composition and electronic-structure modulation. The survey spectra (Figure 4a, 0–1200 eV) show characteristic RE 4d features: Ce 4d (108–112 eV), Sm 4d (134–138 eV), Er 4d (168–172 eV), Tm 4d (175–180 eV), and Yb 4d (185–190 eV) [46,47]. These features support the introduction of RE species, while their low intensity is consistent with low apparent RE contents. Pristine CuO shows only Cu 2p (~930 eV), O 1s (~530 eV), and adventitious carbon C 1s (~285 eV) signals.

High-resolution Cu 2p spectra (Figure 4b) provide insight into Cu oxidation states and their modulation after RE modification. Pristine CuO shows a Cu^+^ component at 932.5 eV, typically associated with Cu_2_O species or partially reduced Cu species, together with a dominant Cu^2+^ peak at 933.8 eV characteristic of monoclinic CuO. The Cu^2+^ state is further supported by distinct satellite peaks at 941.5 and 943.8 eV in the Cu 2p_3_/_2_ region and at 962.3 eV in the Cu 2p_1_/_2_ region [47]. After RE modification, subtle shifts and intensity variations in Cu 2p peaks and satellite structures are observed, suggesting changes in the Cu^+^/Cu^2+^ ratio and local coordination environment. These variations are attributed to RE-associated structural perturbation and defect-related local oxygen environments rather than direct proof of lattice substitution.

The O 1s spectra (Figure 4c) show significant evolution in oxygen environments. Pristine CuO displays a dominant lattice oxygen peak at 529.7 eV, corresponding to O^2−^ species in the Cu–O framework [47]. A weaker high-binding-energy component is observed in pristine CuO and becomes more pronounced after RE modification. The component centered at approximately 531.3 eV may contain overlapping contributions from low-coordination oxygen, surface hydroxyl groups, adsorbed oxygen species, and other defect-associated surface environments. Because these contributions cannot be unequivocally separated by O 1s XPS alone, this component is not assigned exclusively to oxygen vacancies. The observed evolution is therefore interpreted conservatively as evidence of modified surface oxygen chemical environments. Such environments may influence surface adsorption and interfacial redox reactivity; however, their specific role in charge trapping is not directly established by the present XPS data.

High-resolution RE 4d spectra (Figure 4d) further support the introduction of the corresponding RE species and reveal element-dependent chemical-state characteristics. The Ce-related spectral features are consistent with the coexistence of Ce species in more than one oxidation state, whereas the Sm-, Er-, Tm-, and Yb-related spectra are predominantly consistent with trivalent RE species [13,46,47]. The Ce-related result provides ex situ surface-chemical evidence for mixed-valence characteristics in Ce-CuO. However, because of the multiplet complexity and overlap of the Ce 4d region, the present spectrum is used for qualitative chemical-state identification rather than quantitative determination of the Ce^3+^/Ce^4+^ ratio.

The Ce-related mixed-valence surface chemistry may provide reversible electron-accepting and electron-donating sites. A plausible pathway involves temporary reduction of Ce^4+^ to Ce^3+^ through electron acceptance, followed by electron transfer from Ce^3+^ to an interfacial acceptor and regeneration of Ce^4+^. Such behavior has been proposed in other Ce-containing oxide photocatalysts and may contribute to the modified surface-oxygen environment and interfacial electronic response of Ce-CuO [16,17,18]. Nevertheless, the present ex situ XPS analysis does not directly demonstrate Ce^3+^/Ce^4+^ interconversion under simulated solar irradiation. The mixed-valence characteristics are therefore interpreted as a possible contributor to the Ce-CuO performance rather than proof of an operating redox cycle.

Combined XRD, XPS, EDS spectra, and EDS mapping results support the introduction of low-level RE-associated species and their influence on the local structural and chemical environments of CuO. The distinct chemical behavior of the RE species, particularly the Ce-related mixed-valence characteristics, may influence the local electronic structure and interfacial reactivity. These structural and surface-chemical modifications provide a possible basis for the different optical, electrochemical, and photocatalytic responses discussed below, although they do not directly demonstrate improved photogenerated charge separation. Unlike previous studies focused on a single substitutional RE composition, the present work compares Ce-, Sm-, Er-, Tm-, and Yb-modified CuO samples prepared and evaluated under identical conditions. The results show that the effects of RE modification are strongly element-dependent: Ce-CuO exhibits the most favorable combined optical, surface-chemical, and interfacial electrochemical characteristics, whereas the other RE-modified samples display different balances among these properties.

### 3.2. Photocatalytic Degradation Performance

Before photocatalytic irradiation, the adsorption behavior of MO on pristine and RE-modified CuO was examined in the dark over 30 min. As shown in Figure S6, all samples exhibited an initial decrease in C_t_/C_initial_ during the first 5 min, followed by only minor changes during the subsequent dark-stirring period. At 5 min, the C_t_/C_initial_ values were 0.95521 for CuO, 0.93420 for Ce-CuO, 0.94320 for Sm-CuO, 0.94147 for Er-CuO, 0.95240 for Tm-CuO, and 0.96000 for Yb-CuO. From 5 to 30 min, the additional changes were limited to 0.00330–0.00504, indicating that adsorption had already approached equilibrium within 5 min under the present experimental conditions. Therefore, the concentration measured after the 5 min dark-treatment period was used as C_0_ for the subsequent photocatalytic analysis.

**Figure 5:**
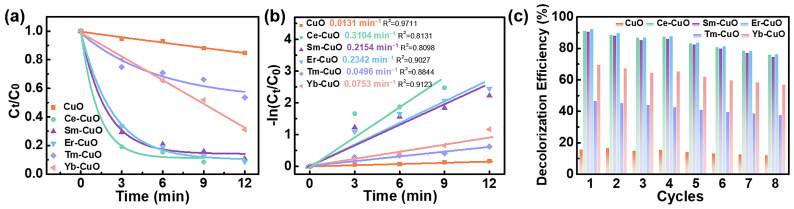
Photocatalytic decolorization performance of pristine and RE-modified copper-oxalate-derived CuO photocatalysts toward methyl orange under simulated solar irradiation: (**a**) representative temporal evolution of C_t_/C_0_, (**b**) corresponding apparent pseudo-first-order kinetic fitting, and (**c**) cycling decolorization performance over eight consecutive cycles. Panels (**a**,**b**) show one representative experiment; complete results from three independent experiments are provided in Figure S7 and Table S4.

Representative temporal decolorization profiles and their corresponding apparent pseudo-first-order fits are shown in Figure 5a,b. The reproducibility of the photocatalytic results was evaluated through three independent experiments for each catalyst. The complete replicate profiles and the corresponding statistical results are presented in Figure S7 and Table S4, respectively. The replicate measurements reproduced the same overall activity trend, and Ce-CuO retained the highest triplicate-mean apparent rate constant under the investigated conditions.

The time-dependent UV–vis absorption spectra are shown in Figure S8. The characteristic absorption band of MO at approximately 463 nm gradually decreased under simulated solar irradiation, demonstrating progressive decolorization and disruption of the MO chromophoric structure. This spectral decrease alone is not interpreted as evidence of complete mineralization. After 12 min of simulated solar irradiation, pristine CuO exhibited only 15.38% MO decolorization, whereas Sm-CuO, Er-CuO, Tm-CuO, and Yb-CuO achieved 89.23%, 91.33%, 46.39%, and 68.86% decolorization, respectively. Ce-CuO reached 91.59% decolorization within 9 min.

In addition to the progressive decrease in the principal MO absorption band at approximately 463 nm, the UV–vis spectra in Figure S8 show a transient absorption feature at 410–430 nm during simulated solar irradiation. The transient feature at 410–430 nm suggests the formation of spectrally distinct transformation intermediates during the early disruption of the MO chromophore. Because UV–vis spectroscopy alone cannot identify these species, a specific N-demethylation pathway is not assigned [48,49]. The feature is more pronounced for Ce-CuO, Sm-CuO, and Er-CuO, indicating more rapid spectral evolution during the early reaction stage. However, the identities of the corresponding transformation products cannot be confirmed from UV–vis spectroscopy alone.

The representative decolorization data were analyzed using the apparent pseudo-first-order kinetic model shown in Figure 5b. The representative k_obs_ values were 0.0131 min^−1^ for pristine CuO, 0.3104 min^−1^ for Ce-CuO, 0.2154 min^−1^ for Sm-CuO, 0.2342 min^−1^ for Er-CuO, 0.0496 min^−1^ for Tm-CuO, and 0.0753 min^−1^ for Yb-CuO. Thus, all RE-modified samples exhibited higher apparent decolorization rate constants than pristine CuO under the fixed experimental conditions. In the triplicate analysis summarized in Table S4, Ce-CuO retained the highest mean k_obs_. Er-CuO exhibited a slightly higher triplicate-mean value than Sm-CuO; however, the corresponding values of 0.2355 ± 0.0236 and 0.2139 ± 0.0189 min^−1^, respectively, showed overlapping mean ± standard-deviation ranges. This modest difference is therefore interpreted cautiously and is not used to establish a uniquely superior intrinsic photocatalytic mechanism for Er-CuO relative to Sm-CuO. The comparatively lower R^2^ ranges obtained for Ce-CuO (0.8131–0.8674) and Sm-CuO (0.7765–0.8098) further indicate that the apparent pseudo-first-order model provides only an approximate description of their rapidly evolving decolorization profiles. The fitted k_obs_ values are consequently used as condition-specific comparative descriptors rather than exact intrinsic kinetic constants. The activity differences are discussed in relation to the combined optical, surface-chemical, apparent carrier-density, and interfacial impedance characteristics, while improved photogenerated charge separation is not treated as directly demonstrated in the absence of recombination-sensitive measurements.

The present photocatalytic experiments were designed to compare the prepared catalysts under fixed physicochemical conditions rather than to optimize the reaction process. All samples were tested at a catalyst concentration of 1.0 g L^−1^ and an initial MO concentration of 10 mg L^−1^, while catalyst loading, solution pH, and initial pollutant concentration were not systematically varied. These variables can influence the apparent kinetics through coupled adsorption, surface-reaction, and optical effects. In particular, although increasing catalyst loading may provide more accessible surface sites, the relatively high solid concentration used here may also promote particle aggregation, suspension turbidity, light scattering, and optical shielding, thereby reducing the effective penetration of incident light. Solution pH can alter catalyst surface charge, MO adsorption behavior, and reactive-species chemistry, whereas increasing the initial MO concentration can enhance competition for accessible surface sites and produce inner-filter effects. Accordingly, the reported k_obs_ values are interpreted as condition-specific comparative descriptors rather than intrinsic or catalyst-loading-optimized kinetic constants. The identical catalyst concentration, solution volume, stirring conditions, and irradiation geometry provide a consistent basis for internal comparison; however, sample-dependent differences in aggregation, morphology, and optical properties mean that variations in light scattering cannot be completely excluded. Systematic loading-, pH-, and concentration-dependent investigations are therefore required for process optimization and remain beyond the scope of the present work.

To determine whether the pronounced spectral decolorization was accompanied by aqueous organic-carbon removal, NPOC analysis was performed for the initial MO solution and the supernatant obtained after Ce-CuO treatment for 9 min under simulated solar irradiation. As shown in Figure S9, the NPOC concentration decreased from 4.94 mg L^−1^ in the initial MO solution to 1.405 mg L^−1^ after treatment, corresponding to an apparent NPOC reduction of 71.6%. This result demonstrates that the disappearance of the characteristic MO absorption band was accompanied by substantial removal of non-purgeable organic carbon from the aqueous phase rather than chromophore decolorization alone. However, the residual NPOC concentration confirms that complete mineralization was not achieved. Moreover, because NPOC analysis does not identify individual transformation products and cannot fully distinguish photocatalytic mineralization from possible adsorption-related organic-carbon removal onto the recovered catalyst, the 71.6% value is interpreted as an apparent aqueous organic-carbon reduction rather than an absolute mineralization efficiency. Identification and toxicity assessment of the transformation products require further investigation.

Figure 5c shows the cycling behavior of the catalysts over eight consecutive photocatalytic runs. Each catalyst was evaluated using the same irradiation duration as its corresponding decolorization profile in Figure 5a: 9 min for Ce-CuO and 12 min for pristine CuO, Sm-CuO, Er-CuO, Tm-CuO, and Yb-CuO. Pristine CuO exhibited consistently low decolorization efficiencies of 11.8–16.4%. In contrast, Ce-CuO and Er-CuO maintained the highest endpoint decolorization efficiencies among the investigated samples. Ce-CuO decreased from 91.2% in the first cycle to 75.8% after eight cycles, corresponding to retention of approximately 83.1% of its cycle-1 efficiency. Er-CuO decreased from 92.1% to 76.3%, corresponding to approximately 82.8% retention, while Sm-CuO decreased from 90.4% to 74.6%, corresponding to approximately 82.5% retention. Yb-CuO and Tm-CuO exhibited lower endpoint efficiencies of 56.8–69.5% and 37.4–46.5%, respectively. Because Ce-CuO and the remaining catalysts were evaluated over different reaction-time ranges, the absolute endpoint efficiencies are used to describe the within-sample cycling evolution and should not be interpreted as a strict common-time stability ranking. Overall, the RE-modified samples retained substantially greater MO decolorization activity than pristine CuO throughout the eight-cycle evaluation, although gradual deactivation was observed. The activity loss may reflect catalyst loss during recovery, surface accumulation of transformation products, changes in aggregation, and/or redistribution of surface-associated RE species.

To further evaluate the post-reaction stability of the best-performing catalyst, Ce-CuO recovered after the eighth photocatalytic cycle was characterized by XRD, SEM, and EDS. As shown in Figure S10a, the principal diffraction features of monoclinic CuO were retained after cycling, and no obvious new crystalline secondary phase was detected. The post-cycling SEM image in Figure S10b shows that the overall aggregated particle morphology remained recognizable, although local changes in particle aggregation and surface texture cannot be excluded. The corresponding EDS spectrum in Figure S10c confirms the continued presence of Cu, O, and Ce. As summarized in Table S5, the local semi-quantitative Cu, O, and Ce contents changed from 37.8, 61.0, and 1.2 at.% in the as-prepared Ce-CuO sample to 31.1, 68.5, and 0.4 at.% after eight cycles, respectively. The lower local Ce signal after cycling may be associated with partial loss or redistribution of Ce-associated species; however, differences between the selected analysis regions and the local semi-quantitative nature of EDS must also be considered. The EDS values are therefore not interpreted as quantitative bulk elemental-retention ratios. Overall, the post-cycling results indicate that the major CuO crystalline phase and the general aggregated morphology were largely retained. The gradual activity decline may be associated with catalyst loss during recovery, surface accumulation of transformation products, and/or partial redistribution of surface-associated Ce species, although the individual contributions of these factors were not independently quantified.

To provide a transparent comparison with previously reported CuO-based photocatalysts, the photocatalytic performance of the present pristine CuO and Ce-CuO samples was compared with representative CuO and CuO-based composite photocatalysts, as summarized in Table 1. The table focuses primarily on MO decolorization or removal studies and includes the irradiation source, reaction time, reported removal efficiency, and apparent pseudo-first-order rate constant where available. The reported performance varies substantially with synthesis route, catalyst composition, morphology, irradiation source, pollutant concentration, catalyst dosage, solution pH, and reaction duration. The reported irradiation periods span 4–300 min, placing the 9 min treatment used for the present Ce-CuO sample toward the short-time end of the comparison without establishing it as the shortest reported treatment. Under the fixed conditions used in this work, Ce-CuO achieved 91.59% MO decolorization within 9 min and exhibited a representative apparent k_obs_ of 0.3104 min^−1^, whereas pristine CuO showed 15.38% decolorization within 12 min and a representative k_obs_ of 0.0131 min^−1^. Because the experimental conditions differ among the listed studies, this comparison provides descriptive short-time kinetic context and is not used to establish an absolute condition-normalized performance ranking.

### 3.3. Optical and Electrochemical Properties

**Figure 6:**
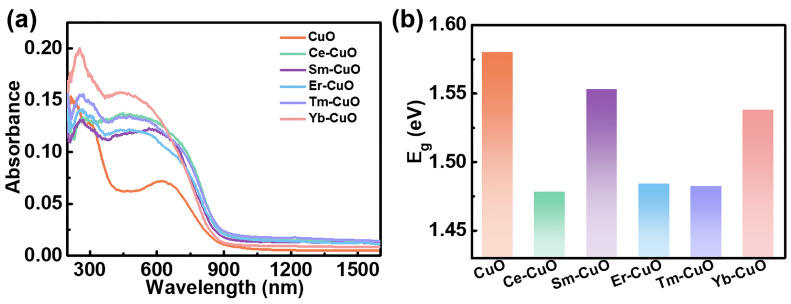
Optical properties of pristine and RE-modified copper-oxalate-derived CuO photocatalysts: (**a**) UV–vis diffuse-reflectance spectra and (**b**) apparent optical band-gap values derived from the corresponding direct-allowed Tauc analyses. Complete Tauc plots and the selected linear extrapolation regions are provided in Figure S11.

The UV–vis diffuse reflectance spectra in Figure 6a reveal noticeable modifications in the optical absorption properties of CuO after RE modification. All RE-modified samples exhibit stronger absorption in the 400–800 nm range compared with pristine CuO. Two key features are observed: (1) a discernible red shift of the absorption edge and (2) enhanced absorption intensity across the visible region, indicating a stronger visible-region optical response. This broad-spectrum absorption enhancement may arise from RE-associated electronic interactions, modified surface chemical environments, and morphology-related optical effects.

The complete direct-allowed Tauc plots and the selected linear extrapolation regions are provided in Figure S11, while Figure 6b summarizes the resulting apparent optical band-gap values. Linear extrapolation to the photon-energy axis yielded E_g_ values of 1.580 eV for pristine CuO, 1.478 eV for Ce-CuO, 1.553 eV for Sm-CuO, 1.484 eV for Er-CuO, 1.482 eV for Tm-CuO, and 1.538 eV for Yb-CuO. Thus, all RE-modified samples exhibited slightly lower apparent band-gap values than pristine CuO, with Ce-CuO showing the largest decrease of 0.102 eV. These modest changes indicate RE-dependent shifts in the apparent optical absorption edge and are consistent with changes in the local chemical and electronic environments of CuO. However, Tauc-derived band-gap values depend on the assumed transition type and the selected linear extrapolation region. They are therefore interpreted as internally consistent comparative estimates rather than exact intrinsic band-gap values. Moreover, the apparent band-gap trend does not fully reproduce the photocatalytic-activity order, indicating that optical absorption alone does not determine the overall photocatalytic performance.

**Figure 7:**
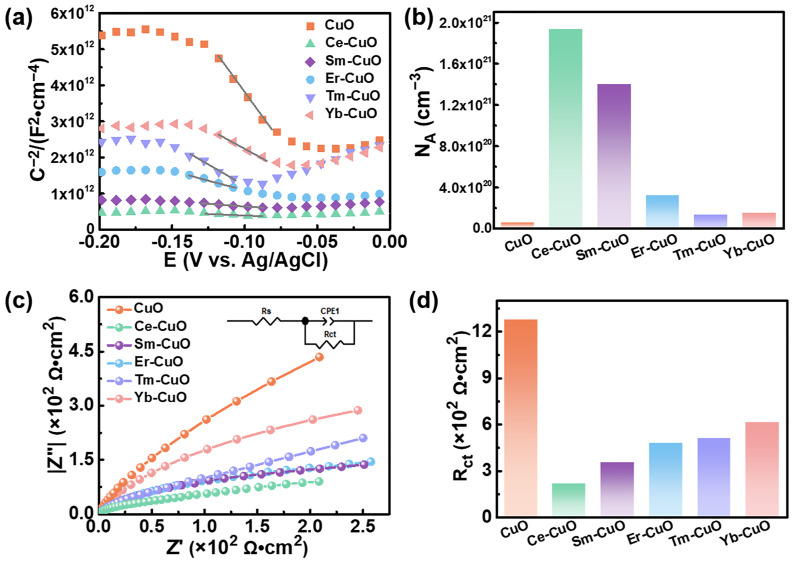
Electrochemical characterization of pristine and RE-modified copper-oxalate-derived CuO photocatalysts: (**a**) Mott–Schottky plots, (**b**) Mott–Schottky-derived apparent majority-carrier-density descriptors, (**c**) EIS Nyquist plots, and (**d**) fitted charge-transfer resistance values.

Mott–Schottky plots (Figure 7a) are consistent with the p-type semiconductor behavior of all samples, as indicated by the negative slopes observed between −0.20 and 0.00 V versus Ag/AgCl. The apparent majority-carrier-density descriptors, expressed as the mean ± fitting-range standard deviation obtained from three adjacent negative-slope fitting windows of each original Mott–Schottky curve, were (5.87 ± 0.23) × 10^19^ cm^−3^ for pristine CuO, (1.94 ± 0.04) × 10^21^ cm^−3^ for Ce-CuO, (1.40 ± 0.14) × 10^21^ cm^−3^ for Sm-CuO, (3.22 ± 0.09) × 10^20^ cm^−3^ for Er-CuO, (1.36 ± 0.02) × 10^20^ cm^−3^ for Tm-CuO, and (1.53 ± 0.04) × 10^20^ cm^−3^ for Yb-CuO. As summarized in Table S6, the corresponding fitting-range relative standard deviations were 3.97%, 1.99%, 9.79%, 2.92%, 1.64%, and 2.83%, respectively. Sm-CuO exhibited the greatest sensitivity to fitting-window selection, whereas the remaining samples showed comparatively smaller variations. These uncertainty values quantify fitting-range sensitivity within a single Mott–Schottky dataset and should not be interpreted as experimental repeatability.

Ce-CuO and Sm-CuO exhibited substantially higher apparent majority-carrier-density descriptors than pristine CuO, whereas Er-CuO, Tm-CuO, and Yb-CuO showed more moderate increases. Nevertheless, these values remain sensitive to the adopted dielectric-constant approximation, electrode geometry, fitting-window selection, measurement frequency, surface roughness, and non-ideal interfacial capacitance. They are therefore interpreted as comparative apparent descriptors rather than exact intrinsic bulk carrier concentrations. Moreover, the apparent N_A_ descriptor does not directly quantify the population, lifetime, separation efficiency, or surface utilization of photogenerated carriers during photocatalysis. Notably, Sm-CuO exhibited a substantially higher apparent N_A_ and a somewhat lower fitted R_ct_ than Er-CuO, while its triplicate-mean k_obs_ was slightly lower. This non-monotonic relationship is not contradictory because N_A_, R_ct_, and k_obs_ describe different aspects of the electrode and photocatalytic reaction system. The Mott–Schottky results are therefore treated as evidence of altered apparent electronic characteristics rather than as a direct predictor of photocatalytic activity.

For the comparative band-edge analysis, a separate representative negative-slope linear fit was selected from each original Mott–Schottky dataset to determine the x-axis intercept and apparent flat-band potential. This fitting procedure was distinct from the three adjacent negative-slope fitting windows used only to evaluate the fitting-range sensitivity of the apparent N_A_ descriptor in Table S6. Consequently, the Mott–Schottky R^2^ values reported in Table S7 correspond to the representative intercept fits and are not expected to fall within the R^2^ ranges reported in Table S6. By combining the resulting apparent E_fb_ values with the Tauc-derived apparent optical band gaps, comparative apparent Fermi-level, valence-band, and conduction-band positions were estimated. The apparent E_F_ values ranged from −0.076 to +0.169 V, the apparent E_VB_ values ranged from +0.024 to +0.269 V, and the apparent E_CB_ values ranged from −1.547 to −1.284 V versus Ag/AgCl. For Ce-CuO, the estimated apparent E_F_, E_VB_, and E_CB_ values were −0.076, +0.024, and −1.454 V versus Ag/AgCl, respectively. The resulting comparative apparent band alignment is illustrated in Figure S12. These positions provide an internally consistent basis for comparing the samples but should not be interpreted as direct absolute energy-level measurements. Their non-monotonic relationship with photocatalytic activity further indicates that the apparent band positions alone do not determine the overall reaction performance.

The electrochemical impedance spectroscopy (EIS) results (Figure 7c,d) provide insight into charge-transfer behavior. The Nyquist plots exhibit semicircular arcs typical of charge-transfer processes at the electrode/electrolyte interface. The fitted charge-transfer resistance (R_ct_) values (Figure 7d) follow the order: Ce-CuO (216 Ω·cm^2^) < Sm-CuO (298 Ω·cm^2^) < Er-CuO (354 Ω·cm^2^) < Tm-CuO (512 Ω·cm^2^) < Yb-CuO (687 Ω·cm^2^) < pristine CuO (1280 Ω·cm^2^). The lower fitted R_ct_ values after RE modification indicate reduced interfacial charge-transfer resistance under the electrochemical measurement conditions. Ce-CuO shows the lowest fitted resistance, representing an approximately 83% reduction compared with pristine CuO and indicating a comparatively favorable interfacial charge-transfer environment. The particularly low fitted R_ct_ of Ce-CuO is consistent with favorable Ce-associated surface electronic interactions and may be partially related to its mixed-valence surface chemistry. Tm-CuO and Yb-CuO also exhibit lower R_ct_ values than pristine CuO, confirming that RE modification changes the interfacial impedance characteristics. However, EIS reflects the combined electrode/electrolyte interfacial response and does not independently demonstrate a Ce^3+^/Ce^4+^ redox pathway, photogenerated-carrier lifetime, charge-separation efficiency, or electron–hole recombination kinetics. Moreover, the photocatalytic activity trend does not strictly follow either R_ct_ or the apparent N_A_ descriptor alone, as particularly illustrated by the comparison between Sm-CuO and Er-CuO. This non-monotonic behavior indicates that activity is governed by the combined optical, interfacial, adsorption-related, and surface-reaction characteristics of the catalyst. In the absence of PL, time-resolved PL, or transient photocurrent measurements, the present electrochemical results are therefore interpreted as indirect evidence of favorable interfacial charge-transfer characteristics rather than direct proof of enhanced photogenerated charge separation or suppressed recombination.

Previous electrochemical studies of rare-earth-containing oxide electrodes provide useful context for the element-dependent impedance behavior observed here. For example, PrCoO_3_, NdCoO_3_, and SmCoO_3_ perovskite electrodes exhibited distinct cyclic-voltammetric and impedance responses that were correlated with differences in rare-earth-dependent structural disorder, electrical conductivity, and electrochemical behavior [21]. In another oxide-electrode system, Ce-containing modified PbO_2_ electrodes showed altered oxidation characteristics in cyclic voltammetry and lower fitted charge-transfer resistance than the corresponding comparison electrodes [22]. These studies support the broader view that lanthanide identity and incorporation mode can influence oxide-electrode surface redox behavior and interfacial charge-transfer characteristics. However, the host oxides, electrolytes, electrode reactions, and modification routes in those studies differ substantially from the present RE-modified CuO photocatalysts. Therefore, they are used only as electrochemical context and not as evidence that the same redox or transport mechanisms operate in the present system. Because cyclic voltammetry was not performed here, changes in surface redox peak positions or redox kinetics are not directly claimed.

### 3.4. Literature-Derived Kinetic Contextualization

**Figure 8:**
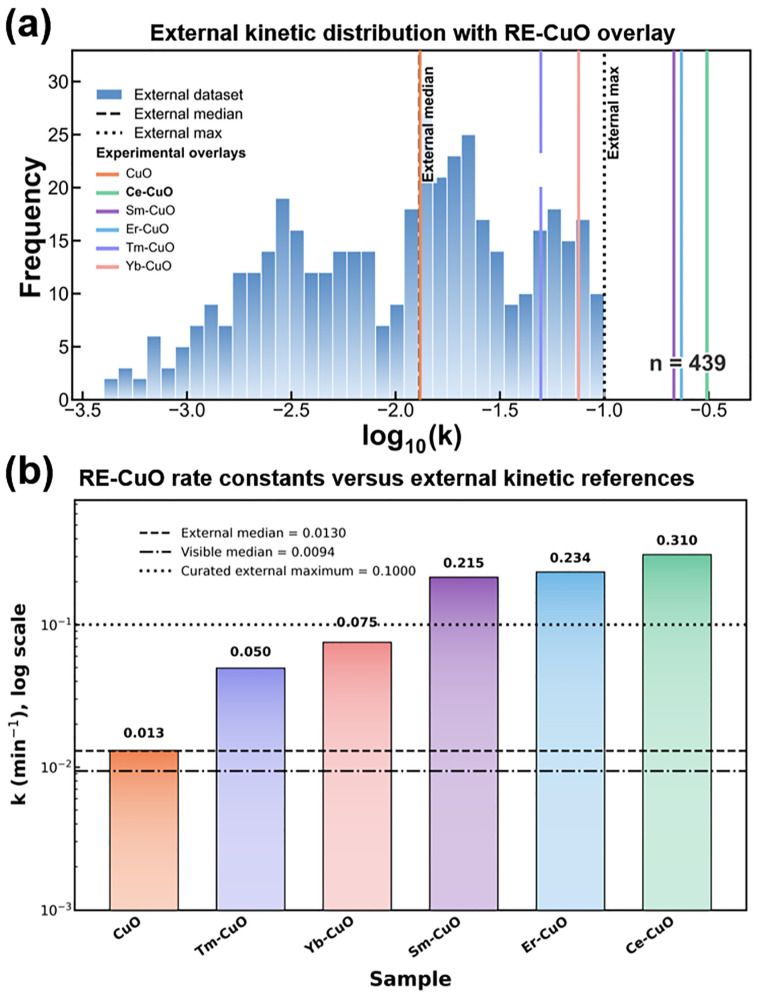
Literature-derived kinetic contextualization of the experimentally measured apparent rate constants of pristine and RE-modified copper-oxalate-derived CuO photocatalysts: (**a**) frequency distribution of log_10_(k) in the curated external photocatalytic-degradation dataset with the experimental values overlaid and (**b**) comparison of the experimental values with the external median, visible-light median, 95th percentile, and curated maximum. Samples in panel (**b**) are arranged in ascending order of the measured k_obs_. Because the literature reaction conditions were not normalized, the comparison provides descriptive external kinetic context only and is not interpreted as a condition-normalized performance ranking.

The experimentally measured apparent rate constants, k_obs_, were compared with a curated external dataset comprising 439 photocatalytic degradation records collected from 54 literature references. The external dataset exhibited a median apparent rate constant of 0.0130 min^−1^, a 95th percentile of 0.0751 min^−1^, and a curated maximum of 0.1000 min^−1^. As shown in Figure 8, pristine CuO (k_obs_ = 0.0131 min^−1^) was located close to the external median. Tm-CuO (0.0496 min^−1^) was positioned above the median, while Yb-CuO (0.0753 min^−1^) was located near the 95th percentile. Sm-CuO (0.2154 min^−1^), Er-CuO (0.2342 min^−1^), and Ce-CuO (0.3104 min^−1^) exceeded the curated maximum of the external reference dataset.

These positions provide only a descriptive external reference for the experimentally measured kinetics. The literature records were obtained under heterogeneous irradiation spectra and intensities, catalyst dosages, pollutant identities and concentrations, pH values, adsorption procedures, and reactor configurations. Therefore, the comparison is not interpreted as a condition-normalized performance ranking or as evidence that the prepared catalysts are intrinsically superior to every catalyst represented in the external dataset. Reference-grouped regression evaluation further showed weak predictive robustness, with the best-performing model yielding a median RMSE of 0.5587 and a median R^2^ of −0.0033. Consequently, the model outputs were not used to predict the performance of the prepared catalysts, support mechanistic interpretation, or independently validate Ce-CuO. The external-dataset summary is provided in Table S8, the dataset construction and preprocessing procedure is detailed in Table S9, the repeated reference-grouped model robustness is summarized in Table S10, and the sample-level kinetic contextualization is provided in Table S11.

To complement the external kinetic contextualization, Figure S13 presents a descriptive experimental descriptor map relating the representative apparent k_obs_ to the inverse fitted charge-transfer resistance, 1/R_ct_, with bubble size representing the corresponding decolorization efficiency. Ce-CuO occupies the region of comparatively high k_obs_ and high 1/R_ct_, consistent with its comparatively low fitted R_ct_. However, the non-monotonic positions of the remaining samples demonstrate that fitted charge-transfer resistance alone does not determine photocatalytic activity. Figure S13 is therefore interpreted only as an internal cross-descriptor visualization and not as a machine-learning prediction, mechanistic proof, or single-parameter causal relationship.

### 3.5. Temperature-Dependent Kinetics and Reactive-Species Analysis

Figure S14a–f show the temperature-dependent photocatalytic decolorization profiles of MO over pristine and RE-modified CuO catalysts in the temperature range of 303–333 K under simulated solar irradiation. The decolorization efficiency increased monotonically with increasing temperature for all samples, indicating that the apparent photocatalytic kinetics were thermally promoted under the investigated conditions. Ce-CuO exhibited the highest activity and achieved near-complete MO decolorization at 333 K within 12 min, whereas pristine CuO showed the lowest activity and comparatively weak temperature dependence.

The apparent pseudo-first-order rate constants (k_obs_), obtained from the linear fitting of −ln(C/C_0_) against irradiation time, increased with increasing temperature. At each investigated temperature, the apparent rate constants generally followed the order Ce-CuO > Er-CuO > Sm-CuO > Yb-CuO > Tm-CuO > pristine CuO, consistent with the corresponding decolorization trends.

**Figure 9:**
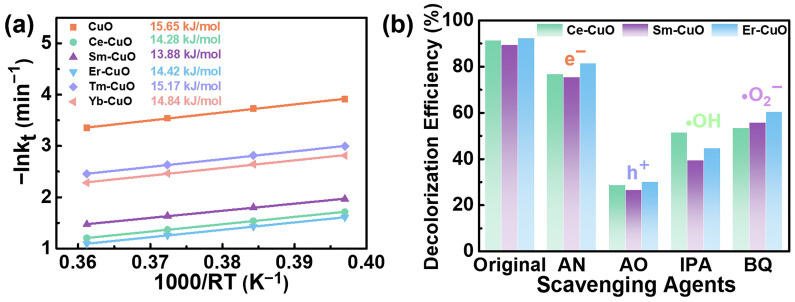
Temperature-dependent apparent kinetic and reactive-species analyses of MO photodecolorization over RE-modified copper-oxalate-derived CuO photocatalysts under simulated solar irradiation: (**a**) Arrhenius plots derived from apparent pseudo-first-order rate constants at different temperatures and (**b**) decolorization efficiencies of Ce-CuO, Sm-CuO, and Er-CuO in the absence and presence of 1 mM AgNO_3_, ammonium oxalate, isopropyl alcohol, or p-benzoquinone.

An Arrhenius plot was constructed by plotting -lnk_T_ as a function of 1000/RT (Figure 9a). The apparent activation energies were calculated to be 15.65 kJ mol^−1^ for pristine CuO, 15.17 kJ mol^−1^ for Tm-CuO, 14.84 kJ mol^−1^ for Yb-CuO, 14.42 kJ mol^−1^ for Er-CuO, 14.28 kJ mol^−1^ for Ce-CuO, and 13.88 kJ mol^−1^ for Sm-CuO.

The relatively narrow E_a_ range indicates that the large differences in photocatalytic activity cannot be explained by the apparent activation barrier alone. Variations in the effective pre-exponential term and in the measured apparent rate constants may reflect the combined influence of light absorption, pollutant adsorption, accessible surface-reaction sites, interfacial charge-transfer characteristics, particle aggregation, and other sample-dependent factors under the fixed reaction conditions. Because photoluminescence, time-resolved photoluminescence, and transient photocurrent measurements were not performed, these kinetic differences are not attributed directly to enhanced photogenerated charge separation or suppressed electron–hole recombination.

Reactive-species involvement was evaluated using AgNO_3_, ammonium oxalate, isopropyl alcohol, and p-benzoquinone as scavengers for electrons, holes, ⋅OH, and ⋅O^2−^, respectively. As shown in Figure 9b, ammonium oxalate produced the strongest inhibition of MO decolorization, followed by isopropyl alcohol and p-benzoquinone, whereas AgNO_3_ showed a comparatively smaller effect. This inhibition pattern suggests that photogenerated holes are the principal oxidative species under the investigated conditions, while ⋅OH and ⋅O_2_^−^ also contribute to MO transformation. Because scavenger molecules may additionally influence adsorption, surface reactions, and interfacial processes, these results are interpreted qualitatively and are not used to quantify the absolute contribution of each reactive species.

### 3.6. Proposed Photocatalytic Mechanism

**Figure 10:**
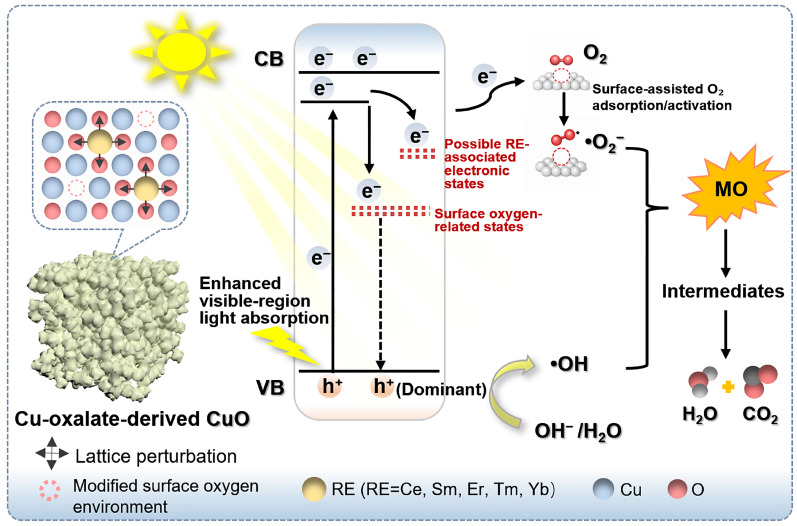
Schematic proposed photocatalytic transformation pathway of MO over RE-modified copper-oxalate-derived CuO under simulated solar irradiation. Black arrows indicate the proposed electron-transfer and reaction pathways; yellow arrows indicate hole-mediated oxidation involving OH^−^/H_2_O; red dashed levels represent the possible RE-associated electronic states and surface oxygen-related states; red dashed circles denote modified surface oxygen environments/possible adsorption sites; and the atomic colors and symbols are defined in the in-figure legend.

On the basis of the combined optical, electrochemical, surface-chemical, kinetic, NPOC, and reactive-species-scavenging results, a conservative photocatalytic transformation pathway is proposed in Figure 10. Under simulated solar irradiation, CuO absorbs incident photons with sufficient energy to generate conduction-band electrons and valence-band holes. RE modification is associated with modest apparent band-gap narrowing, altered surface oxygen chemical environments, changes in the apparent majority-carrier-density descriptors, and reduced fitted interfacial charge-transfer resistance relative to pristine CuO. Possible RE-associated electronic states and surface oxygen-related states may influence the transient accommodation and interfacial transfer of photogenerated electrons. However, because photoluminescence, time-resolved photoluminescence, transient photocurrent, and operando spectroscopic measurements were not performed, these states are represented qualitatively in Figure 10 and are not treated as directly demonstrated electron-trapping centers.

The scavenger experiments showed that ammonium oxalate produced the strongest inhibition of MO decolorization, indicating that photogenerated holes were the principal oxidative species under the investigated conditions. Isopropyl alcohol and p-benzoquinone also inhibited the reaction, suggesting the participation of ·OH and ·O_2_^−^, respectively, whereas AgNO_3_ produced a comparatively smaller effect. Photogenerated holes may therefore directly oxidize adsorbed MO molecules, while conduction-band electrons may be transferred to surface-adsorbed O_2_ to generate ·O_2_^−^. In addition, the participation of ·OH is represented in Equation (20), which is consistent with hole-driven oxidation involving surface OH^−^/H_2_O under the present reaction conditions. Taken together, the scavenger results support a mechanism in which h^+^ plays the dominant role, while ·OH and ·O_2_^−^ act as additional reactive oxidative species that promote the transformation of MO.

For Ce-CuO, the ex situ XPS results are consistent with the coexistence of Ce^3+^- and Ce^4+^-related surface states. Previous studies of Ce-containing oxide photocatalysts provide precedent for Ce^4+^/Ce^3+^ electron exchange and subsequent transfer of an electron to adsorbed O_2_ [16,17,18,55]. Moreover, a previous Ce-loaded CuO study also proposed the Ce^4+^/Ce^3+^-assisted O_2_-activation route, providing direct literature precedent in a compositionally related CuO-based system [31]. On this basis, Ce^4+^ may temporarily accept an electron to form Ce^3+^, followed by electron transfer from Ce^3+^ to adsorbed O_2_ and regeneration of Ce^4+^. Earlier Ce-modified CuO studies support composition-dependent changes in optical and photocatalytic behavior [9,31], but they do not establish that the identical operando Ce cycle occurs in the present material. The proposed electron-exchange sequence could facilitate interfacial O_2_ activation; however, dynamic Ce^3+^/Ce^4+^ interconversion was not monitored under irradiation and is therefore considered a literature-supported mechanistic possibility rather than a directly demonstrated elementary process.

The general photoexcitation and oxygen-reduction steps can be summarized as follows [8,17,18]:

(18) $$CuO+h\nu \to e^{-}+h^{+}$$

(19) $$O_{2(ads)}+e^{-}\to \cdot O_{2}^{-}$$

(20) $$h^{+}+{OH}^{-}/H_{2}O\to \cdot OH+H^{+}$$

For the proposed Ce-related electron-exchange pathway in Ce-CuO, based on the Ce^4+^/Ce^3+^ redox/O_2_-activation precedent reported previously [31,55],

(21) $${Ce}^{4+}+e^{-}\to {Ce}^{3+}$$

(22) $${Ce}^{3+}+O_{2(ads)}\to {Ce}^{4+}+\cdot O_{2}^{-}$$

Further oxidation may produce a mixture of residual organic intermediates and mineralized products [48,49]:

(23) $$Dye+h^{+}/\cdot OH/\cdot O_{2}^{-}\to Intermediates\to {CO}_{2}+H_{2}O$$

In the present experiment, the progressive decrease in the principal MO absorption band and the transient spectral feature at 410–430 nm in Figure S8 provide direct spectral evidence for disruption of the MO chromophore and the temporary presence of spectrally distinct transformation intermediates. Prior MO-transformation studies show that changes in the characteristic UV–vis bands can accompany chromophore cleavage and intermediate formation, whereas assignment of individual products requires chromatographic or mass-spectrometric evidence [48,49,56]. A recent related study employed high-resolution mass spectrometry together with in silico toxicity assessment for product identification and residual-risk evaluation [56], underscoring that decolorization alone does not necessarily demonstrate complete detoxification. Because the intermediates in the present work were not identified by chromatographic or mass-spectrometric analysis, a specific molecular degradation sequence is not assigned. As shown in Figure S9, the NPOC concentration decreased from 4.94 to 1.405 mg L^−1^ after 9 min of Ce-CuO treatment, corresponding to an apparent aqueous organic-carbon reduction of 71.6%. This result indicates that the pronounced spectral decolorization was accompanied by substantial organic-carbon removal and deep oxidative transformation rather than simple chromophore bleaching alone. At the same time, the residual NPOC shows that mineralization was not complete within the tested reaction period. Therefore, the mechanistic pathway shown in Figure 10 and Equations (18)–(23) is interpreted as reflecting substantial transformation of MO into intermediate species together with partial conversion to mineralized products. Accordingly, the CO_2_ and H_2_O shown in Figure 10 and Equation (23) represent possible products of the mineralized fraction rather than complete conversion of all MO-derived carbon.

Overall, the superior performance of Ce-CuO is interpreted as arising from the combined influence of its visible-region optical response, modified surface oxygen environment, apparent majority-carrier-density characteristics, and comparatively low fitted interfacial charge-transfer resistance. Within this framework, the dominant contribution of photogenerated holes, the auxiliary participation of ·OH and ·O_2_^−^, and the possible Ce^4+^/Ce^3+^-mediated O_2_ activation pathway together provide a reasonable explanation for the enhanced short-time MO decolorization observed for Ce-CuO. Possible RE-associated electronic states and Ce^3+^/Ce^4+^ electron exchange may contribute to interfacial electron transfer and O_2_ activation, but neither process is presented as independently demonstrated. The proposed mechanism therefore describes experimentally supported reactive-species involvement and conservative literature-supported interfacial pathways without assigning the observed activity to a single unverified elementary mechanism.

## 4. Conclusions

In summary, RE-modified copper-oxalate-derived CuO nanostructures were prepared, and the relationships among RE identity, apparent structural perturbation, surface chemistry, optical response, electrochemical characteristics, and photocatalytic behavior were systematically examined. RE modification was accompanied by modest changes in interplanar spacing, surface oxygen environments, apparent optical band gaps, Mott–Schottky-derived majority-carrier-density descriptors, and fitted interfacial charge-transfer resistance. Among the investigated samples, Ce-CuO exhibited the highest photocatalytic activity, achieving 91.59% MO decolorization within 9 min and a representative apparent k_obs_ of 0.3104 min^−1^. Its NPOC concentration decreased from 4.94 to 1.405 mg L^−1^ after 9 min, corresponding to an apparent aqueous organic-carbon reduction of 71.6%; however, the residual NPOC confirmed that complete mineralization was not achieved. The non-monotonic relationships among apparent N_A_, fitted R_ct_, and k_obs_, particularly for Sm-CuO and Er-CuO, demonstrate that no single optical or electrochemical descriptor independently governs the overall photocatalytic performance. Scavenger experiments indicated that h^+^ was the principal oxidative species, while ·OH and ·O_2_^−^ also contributed to MO transformation. Possible RE-associated electronic states and Ce^3+^/Ce^4+^ electron exchange may contribute to interfacial electron transfer and O_2_ activation, but these processes were not directly monitored under irradiation and are therefore treated as mechanistic possibilities. After eight cycles, Ce-CuO retained a decolorization efficiency of 75.8%, corresponding to approximately 83.1% of its cycle-1 value. Post-cycling characterization showed that the principal CuO crystalline phase and overall aggregated morphology were largely retained, although the local semi-quantitative Ce signal decreased. The literature-derived kinetic dataset provided descriptive external context only and was not used as predictive or mechanistic evidence. Overall, the results show that RE modification provides a practical route for regulating the combined structural, surface-chemical, optical, and interfacial characteristics of copper-oxalate-derived CuO photocatalysts.

## Figures and Tables

**Table 1:** Comparison of the photocatalytic performance of the present CuO and Ce-CuO catalysts with representative CuO-based photocatalysts reported in the literature.

Photocatalyst	Pollutant	Irradiation Source	Time (min)	Reported Removal/ Decolorization (%)	k_obs_ (min^−1^)	Ref.
Green-synthesized CuO NPs	MO	UV light	24	96.0	0.1341 ^b^	[26]
Green-synthesized CuO NPs	MO	UV light	24	96.4	0.1385 ^b^	[27]
CuO NPs prepared by chemical precipitation	MO	UV light	120	90.0	0.0192 ^b^	[28]
Green-synthesized CuO NPs	MO	Sunlight	60	95.0	0.0499 ^b^	[29]
Hydrothermally prepared CuO nanorods	MO	Sunlight	90	22.0	0.0028 ^b^	[30]
1 wt.% Ce-loaded CuO nanoparticles	MO	UV-A irradiation	300	95%	0.075 ^a^	[31]
CuO/NC nanocomposite	MO	Visible light	4	95.96	0.8022 ^b^	[50]
ZnO–CuO nanocomposite	MO	UV irradiation	120	92.18	0.0212 ^b^	[51]
CuO/TiO_2_ composite	MO	Visible light	180	92.2	0.0142 ^b^	[52]
1 wt.% Mn + 0.5 wt.% Co co-doped CuO thin film	MO	Visible light	120	87.0	0.0170 ^b^	[53]
Biogenic mechanochemical CuO NPs	MO	Sunlight	60	65.078	0.01753 ^a^	[54]
CuO	MO	Simulated solar irradiation	12	15.38	0.0131	This work
Ce-CuO	MO	Simulated solar irradiation	9	91.59	0.3104	This work

^a^ Reported by the corresponding study based on kinetic fitting. ^b^ Calculated in the present comparison from the reported final decolorization efficiency (η) and irradiation time (t) using k_calc_ = −ln(1 − η/100)/t, assuming apparent pseudo-first-order behavior. These endpoint-derived values are approximate estimates rather than multipoint regression results.

## Data Availability

The original contributions presented in this study are included in the article/Supplementary Materials. Further inquiries can be directed to the corresponding author. No new dataset was created for the machine-learning-assisted kinetic contextualization in this study. The external photocatalysis dataset used for this analysis was obtained from the publicly available Supplementary Materials of Jiang et al. [40].

## Supplementary Materials

The following supporting information can be downloaded at: https://www.mdpi.com/article/10.3390/nano16150908/s1, Figure S1, CCharacterization of the as-prepared copper oxalate precursor: (a) X-ray diffraction (XRD) pattern and (b) scanning electron microscopy (SEM) image; Figure S2, EDS spectra of (a) CuO, (b) Ce-CuO, (c) Sm-CuO, (d) Er-CuO, (e) Tm-CuO, and (f) Yb-CuO, showing Cu and O signals together with detectable low-level RE signals in the corresponding modified samples; Table S1, Semi-quantitative EDS elemental compositions of pristine and RE-modified copper-oxalate-derived CuO samples; Figure S3, EDS elemental mapping of as-prepared Ce-CuO: (a) SEM image, (b) merged elemental map, and individual elemental distributions of (c) Ce, (d) Cu, and (e) O; Table S2, Diffraction peak positions, interplanar spacings, and apparent lattice-perturbation magnitudes of pristine and RE-modified copper-oxalate-derived CuO calculated from the (−111) and (111) planes; Figure S4, XRD peak-broadening-derived structural parameters of pristine and RE-modified CuO: (a) comparison of the apparent Scherrer crystallite size D_S_ and Williamson–Hall crystallite size D_WH_, and (b) signed apparent Williamson–Hall microstrain ε_WH_.; Figure S5, Williamson–Hall plots of (a) CuO, (b) Ce-CuO, (c) Sm-CuO, (d) Er-CuO, (e) Tm-CuO, and (f) Yb-CuO based on five selected monoclinic CuO reflections. Because no instrumental-broadening correction was applied and the fitting linearity was limited for several samples, the resulting parameters are treated as comparative apparent estimates; Table S3, Apparent crystallite sizes and signed Williamson–Hall microstrain parameters of pristine and RE-modified CuO.; Figure S6, Dark-adsorption profiles of methyl orange over pristine and RE-modified CuO during 30 min of dark stirring, expressed as C_t_/C_initial_; Figure S7, Results of three independent photocatalytic decolorization experiments for (a) CuO, (b) Ce-CuO, (c) Sm-CuO, (d) Er-CuO, (e) Tm-CuO, and (f) Yb-CuO under otherwise identical simulated-solar-irradiation conditions; Table S4, Replicate decolorization efficiencies and apparent pseudo-first-order kinetic parameters; Figure S8, Time-dependent UV–vis absorption spectra of methyl orange solution during photocatalytic treatment over (a) CuO, (b) Ce-CuO, (c) Sm-CuO, (d) Er-CuO, (e) Tm-CuO, and (f) Yb-CuO under simulated solar irradiation; Figure S9, NPOC concentrations of the initial MO solution and the Ce-CuO-treated supernatant after simulated-solar photocatalytic treatment; Figure S10, Post-cycling characterization of Ce-CuO recovered after eight consecutive photocatalytic cycles: (a) comparison of the XRD patterns of as-prepared and cycled Ce-CuO, (b) SEM image of the recovered catalyst, and (c) corresponding EDS spectrum; Table S5, Local semi-quantitative EDS elemental compositions of as-prepared Ce-CuO and Ce-CuO recovered after eight photocatalytic cycles; Figure S11, Complete direct-allowed Tauc plots of (a) CuO, (b) Ce-CuO, (c) Sm-CuO, (d) Er-CuO, (e) Tm-CuO, and (f) Yb-CuO; Table S6, Fitting-range sensitivity of the Mott–Schottky-derived apparent majority-carrier-density descriptors; Figure S12, Comparative apparent conduction-band, valence-band, and Fermi-level positions of pristine and RE-modified CuO estimated by combining Mott–Schottky and Tauc analyses. All potentials are reported versus Ag/AgCl. The displayed values are comparative apparent estimates rather than direct absolute energy-level measurements; Table S7, Apparent optical band-gap, flat-band/Fermi-level, valence-band, and conduction-band positions of pristine and RE-modified CuO estimated from Mott–Schottky and Tauc analyses; Figure S13, Descriptive relationship between the representative apparent pseudo-first-order rate constant k_obs_ and inverse fitted charge-transfer resistance 1/R_ct_ for pristine and RE-modified CuO. Bubble size represents the corresponding decolorization efficiency. The plot is used only to visualize the experimental descriptors and does not imply a single-parameter causal relationship between R_ct_ and photocatalytic activity; Table S8, Summary of the curated external photocatalytic kinetic dataset and descriptive benchmarking metrics; Table S9, Construction and preprocessing of the curated external photocatalytic kinetic dataset used for literature-derived machine-learning-assisted contextualization; Table S10, Repeated reference-grouped machine-learning model robustness; Table S11, Detailed machine-learning-assisted kinetic contextualization of CuO and RE-CuO samples; and Figure S14, Temperature-dependent photocatalytic decolorization profiles of methyl orange (MO) over (a) CuO, (b) Ce-CuO, (c) Sm-CuO, (d) Er-CuO, (e) Tm-CuO, and (f) Yb-CuO at 303–333 K under simulated solar irradiation, expressed as C_t_/C_0_ versus reaction time.

## References

[1] Naidu R., Biswas B., Willett I.R., Cribb J., Singh B.K., Nathanail C.P., Coulon F., Semple K.T., Jones K.C., Barclay A. (2021). Chemical Pollution: A Growing Peril and Potential Catastrophic Risk to Humanity. Environ. Int..

[2] Al-Tohamy R., Ali S.S., Li F., Okasha K.M., Mahmoud Y.A.-G., Elsamahy T., Jiao H., Fu Y., Sun J. (2022). A Critical Review on the Treatment of Dye-Containing Wastewater: Ecotoxicological and Health Concerns of Textile Dyes and Possible Remediation Approaches for Environmental Safety. Ecotoxicol. Environ. Saf..

[3] Samarasinghe L.V., Muthukumaran S., Baskaran K. (2024). Recent Advances in Visible Light-Activated Photocatalysts for Degradation of Dyes: A Comprehensive Review. Chemosphere.

[4] Khan H., Shah M.U.H. (2023). Modification Strategies of TiO_2_-Based Photocatalysts for Enhanced Visible Light Activity and Energy Storage Ability: A Review. J. Environ. Chem. Eng..

[5] Ahmad I., Bousbih R., Mahal A., Khan W.Q., Aljohani M., Amin M.A., Jafar N.N.A., Jabir M.S., Majdi H., Alshomrany A.S. (2024). Recent Progress in ZnO-Based Heterostructured Photocatalysts: A Review. Mater. Sci. Semicond. Process..

[6] Shabna S., Dhas S.S.J., Biju C.S. (2023). Potential Progress in SnO_2_ Nanostructures for Enhancing Photocatalytic Degradation of Organic Pollutants. Catal. Commun..

[7] Kute A.D., Gaikwad R.P., Warkad I.R., Gawande M.B. (2022). A Review on the Synthesis and Applications of Sustainable Copper-Based Nanomaterials. Green Chem..

[8] Sibhatu A.K., Weldegebrieal G.K., Sagadevan S., Tran N.N., Hessel V. (2022). Photocatalytic Activity of CuO Nanoparticles for Organic and Inorganic Pollutants Removal in Wastewater Remediation. Chemosphere.

[9] Islam M.R., Saiduzzaman M., Nishat S.S., Kabir A., Farhad S.F.U. (2021). Synthesis, Characterization and Visible Light-Responsive Photocatalysis Properties of Ce-Doped CuO Nanoparticles: A Combined Experimental and DFT+U Study. Colloids Surf. A Physicochem. Eng. Asp..

[10] Chen S., Huang D., Xu P., Xue W., Lei L., Cheng M., Wang R., Liu X., Deng R. (2020). Semiconductor-Based Photocatalysts for Photocatalytic and Photoelectrochemical Water Splitting: Will We Stop with Photocorrosion?. J. Mater. Chem. A.

[11] Xia C., Wang H., Kim J.K., Wang J. (2021). Rational Design of Metal Oxide-Based Heterostructure for Efficient Photocatalytic and Photoelectrochemical Systems. Adv. Funct. Mater..

[12] Jia Z., Yue L., Zheng Y., Xu Z. (2008). The Convenient Preparation of Porous CuO via Copper Oxalate Precursor. Mater. Res. Bull..

[13] Zheng B., Fan J., Chen B., Qin X., Wang J., Wang F., Deng R., Liu X. (2022). Rare-Earth Doping in Nanostructured Inorganic Materials. Chem. Rev..

[14] Wang B., Liu J., Yao S., Liu F., Li Y., He J., Lin Z., Huang F., Liu C., Wang M. (2021). Vacancy Engineering in Nanostructured Semiconductors for Enhancing Photocatalysis. J. Mater. Chem. A.

[15] Apostolova I., Apostolov A., Wesselinowa J. (2022). Band Gap Tuning in Transition Metal and Rare-Earth-Ion-Doped TiO_2_, CeO_2_, and SnO_2_ Nanoparticles. Nanomaterials.

[16] Xu Y., Zhou Y., Li Y., Liu Y., Ding Z. (2024). Advances in Cerium Dioxide Nanomaterials: Synthesis Strategies, Property Modulation, and Multifunctional Applications. J. Environ. Chem. Eng..

[17] Montini T., Melchionna M., Monai M., Fornasiero P. (2016). Fundamentals and Catalytic Applications of CeO_2_-Based Materials. Chem. Rev..

[18] Vento-Lujano E., González L.A. (2021). Defect-Induced Modification of Band Structure by the Insertion of Ce^3+^ and Ce^4+^ in SrTiO_3_: A High-Performance Sunlight-Driven Photocatalyst. Appl. Surf. Sci..

[19] Barrocas B., Sério S., Rovisco A., Melo Jorge M.E. (2014). Visible-Light Photocatalysis in Ca_0.6_Ho_0.4_MnO_3_ Films Deposited by RF-Magnetron Sputtering Using Nanosized Powder Compacted Target. J. Phys. Chem. C.

[20] Tian N., Zhang Y., Huang H., He Y., Guo Y. (2014). Influences of Gd Substitution on the Crystal Structure and Visible-Light-Driven Photocatalytic Performance of Bi_2_WO_6_. J. Phys. Chem. C.

[21] Tripathi H.S., Karmakar R., Bhowmik T.K., Halder S., Dutta A., Sinha T.P. (2022). RCoO_3_ (R = Pr, Nd and Sm) Electrode-Based for Efficient Solid-State Symmetric Supercapacitor. Solid State Sci..

[22] Liu S., Gui L., Peng R., Yu P. (2020). A Novel Porous Ni, Ce-Doped PbO_2_ Electrode for Efficient Treatment of Chloride Ion in Wastewater. Processes.

[23] Hu Y., Pan Y., Wang Z., Lin T., Gao Y., Luo B., Hu H., Fan F., Liu G., Wang L. (2020). Lattice Distortion Induced Internal Electric Field in TiO_2_ Photoelectrode for Efficient Charge Separation and Transfer. Nat. Commun..

[24] Ahmed M.A., Mohamed A.A. (2024). Advances in Ultrasound-Assisted Synthesis of Photocatalysts and Sonophotocatalytic Processes: A Review. iScience.

[25] Pandiyarajan T., Saravanan R., Karthikeyan B., Gracia F., Mansilla H.D., Gracia-Pinilla M.A., Mangalaraja R.V. (2017). Sonochemical Synthesis of CuO Nanostructures and Their Morphology Dependent Optical and Visible Light Driven Photocatalytic Properties. J. Mater. Sci. Mater. Electron..

[26] Sharma S., Kumar K. (2021). Aloe-Vera Leaf Extract as a Green Agent for the Synthesis of CuO Nanoparticles Inactivating Bacterial Pathogens and Dye. J. Dispers. Sci. Technol..

[27] Sharma S., Kumar K., Thakur N., Chauhan S., Chauhan M.S. (2021). Eco-Friendly Ocimum tenuiflorum Green Route Synthesis of CuO Nanoparticles: Characterizations on Photocatalytic and Antibacterial Activities. J. Environ. Chem. Eng..

[28] Pourmortazavi S.M., Rahimi-Nasrabadi M., Ahmadi F., Ganjali M.R. (2018). CuCO_3_ and CuO Nanoparticles; Facile Preparation and Evaluation as Photocatalysts. J. Mater. Sci. Mater. Electron..

[29] Kayalvizhi S., Sengottaiyan A., Selvankumar T., Senthilkumar B., Sudhakar C., Selvam K. (2020). Eco-Friendly Cost-Effective Approach for Synthesis of Copper Oxide Nanoparticles for Enhanced Photocatalytic Performance. Optik.

[30] Sali R.K., Pujar M.S., Nadgir A., Sidarai A.H. (2020). Photocatalytic Activities of CuO Nanorods over Methyl Orange: Hydrothermal Method. AIP Conf. Proc..

[31] Sasikala R., Karthikeyan K., Easwaramoorthy D., Bilal I.M., Rani S.K. (2016). Photocatalytic Degradation of Trypan Blue and Methyl Orange Azo Dyes by Cerium Loaded CuO Nanoparticles. Environ. Nanotechnol. Monit. Manag..

[32] Patterson A.L. (1939). The Scherrer Formula for X-Ray Particle Size Determination. Phys. Rev..

[33] Williamson G.K., Hall W.H. (1953). X-Ray Line Broadening from Filed Aluminium and Wolfram. Acta Metall..

[34] Tauc J., Grigorovici R., Vancu A. (1966). Optical Properties and Electronic Structure of Amorphous Germanium. Phys. Status Solidi B.

[35] Gelderman K., Lee L., Donne S.W. (2007). Flat-Band Potential of a Semiconductor: Using the Mott–Schottky Equation. J. Chem. Educ..

[36] Lee S.F., Jimenez-Relinque E., Martinez I., Castellote M. (2023). Effects of Mott–Schottky Frequency Selection and Other Controlling Factors on Flat-Band Potential and Band-Edge Position Determination of TiO_2_. Catalysts.

[37] Sivula K. (2021). Mott–Schottky Analysis of Photoelectrodes: Sanity Checks Are Needed. ACS Energy Lett..

[38] Choi Y.-H. (2019). Effect of the Calcination Temperature and Li(I) Doping on Ethanol Sensing Properties in p-Type CuO Thin Films. Korean J. Mater. Res..

[39] Kyesmen P.I., Nombona N., Diale M. (2021). A Promising Three-Step Heat Treatment Process for Preparing CuO Films for Photocatalytic Hydrogen Evolution from Water. ACS Omega.

[40] Jiang Z., Hu J., Tong M., Samia A.C.S., Zhang H., Yu X. (2021). A Novel Machine Learning Model to Predict the Photo-Degradation Performance of Different Photocatalysts on a Variety of Water Contaminants. Catalysts.

[41] Tavakoli Joorabi F., Kamali M., Sheibani S. (2022). Effect of Aqueous Inorganic Anions on the Photocatalytic Activity of CuO–Cu_2_O Nanocomposite on MB and MO Dyes Degradation. Mater. Sci. Semicond. Process..

[42] Bayat F., Sheibani S. (2022). Enhancement of Photocatalytic Activity of CuO–Cu_2_O Heterostructures Through the Controlled Content of Cu_2_O. Mater. Res. Bull..

[43] Pandiyarajan T., Mangalaraja R.V., Karthikeyan B., Udayabhaskar R., Contreras D., Sepulveda-Guzman S., Gracia-Pinilla M.A. (2022). Influence of RE (Pr^3+^, Er^3+^, Nd^3+^) Doping on Structural, Vibrational and Enhanced Persistent Photocatalytic Properties of ZnO Nanostructures. Spectrochim. Acta A Mol. Biomol. Spectrosc..

[44] Nazir Kayani Z., Chaudhry W., Sagheer R., Riaz S., Naseem S. (2022). Effect of Ce Doping on Crystallite Size, Band Gap, Dielectric and Antibacterial Properties of Photocatalyst Copper Oxide Nano-Structured Thin Films. Mater. Sci. Eng. B.

[45] Singh S.J., Lim Y.Y., Hmar J.J.L., Chinnamuthu P. (2022). Temperature Dependency on Ce-Doped CuO Nanoparticles: A Comparative Study via XRD Line Broadening Analysis. Appl. Phys. A.

[46] Maslakov K.I., Teterin Y.A., Popel A.J., Teterin A.Y., Ivanov K.E., Kalmykov S.N., Petrov V.G., Petrov P.K., Farnan I. (2018). XPS Study of Ion Irradiated and Unirradiated CeO_2_ Bulk and Thin Film Samples. Appl. Surf. Sci..

[47] Moulder J.F., Stickle W.F., Sobol P.E., Bomben K.D. (1992). Handbook of X-Ray Photoelectron Spectroscopy.

[48] Chen Y.P., Liu S.Y., Yu H.Q., Yin H., Li Q.R. (2008). Radiation-Induced Degradation of Methyl Orange in Aqueous Solutions. Chemosphere.

[49] Xie S., Huang P., Kruzic J.J., Zeng X., Qian H. (2016). A Highly Efficient Degradation Mechanism of Methyl Orange Using Fe-Based Metallic Glass Powders. Sci. Rep..

[50] Khan I., Khan I., Usman M., Imran M., Saeed K. (2020). Nanoclay-Mediated Photocatalytic Activity Enhancement of Copper Oxide Nanoparticles for Enhanced Methyl Orange Photodegradation. J. Mater. Sci. Mater. Electron..

[51] Gerawork M. (2020). Photodegradation of Methyl Orange Dye by Using Zinc Oxide–Copper Oxide Nanocomposite. Optik.

[52] Gebremedhin K.H., Gebreegziabher H.G., Weldegebrieal G.K., Ali Y.M. (2025). Synergistic Adsorption–Photocatalysis Effect of CuO/TiO_2_ Composite for High-Efficient Degradation of Methyl Orange. Discov. Nano.

[53] Ria F.S., Bashar M.S., Rahman M.A., Rahman M.K. (2026). Synthesis of Mn–Co Co-Doped CuO Thin Films for Enhanced Photocatalytic Activity under Visible-Light Irradiation. Ceram. Int..

[54] Aroob S., Carabineiro S.A.C., Taj M.B., Bibi I., Raheel A., Javed T., Yahya R., Alelwani W., Verpoort F., Kamwilaisak K. (2023). Green Synthesis and Photocatalytic Dye Degradation Activity of CuO Nanoparticles. Catalysts.

[55] Sun F., Song J., Wen H., Cao X., Zhao F., Qin J., Mao W., Tang X., Dong L., Long Y. (2023). Ce^4+^/Ce^3+^ Redox Effect-Promoted CdS/CeO_2_ Heterojunction Photocatalyst for the Atom Economic Synthesis of Imines under Visible Light. Inorg. Chem..

[56] Vale M., Barrocas B.T., Serôdio R.M.N., Oliveira M.C., Lopes J.M., Marques A.C. (2024). Robust Photocatalytic MICROSCAFS^®^ with Interconnected Macropores for Sustainable Solar-Driven Water Purification. Int. J. Mol. Sci..

