# Supplementary material for: Rare-Earth-Modified Copper-Oxalate-Derived CuO Nanostructures for Rapid Methyl Orange Photodegradation Under Simulated Solar Irradiation
Source: Nanomaterials (Basel). 2026 Jul 24;16(15):908. doi: 10.3390/nano16150908 (PMC13468492; doi:10.3390/nano16150908)
Supplement: Supplementary file 1 [file nanomaterials-16-00908-s001.zip › nanomaterials-4432328-supplementary.pdf]

Rare-Earth-Modified Copper-Oxalate-Derived CuO Nanostructures for Rapid Methyl Orange Photodegradation Under Simulated Solar Irradiation

Hangning Wang [1], Rifath Bin Hossain [2], Yanling Yang [3], Mengran Wu [1], Xinyu Dai [1] and Fengxiang Qin [1,*]

[1] School of Materials Science and Engineering, Nanjing University of Science and Technology,
Nanjing 210094, China; whn@njust.edu.cn (H.W.);
124116011402@njust.edu.cn (M.W.); xinyudai@njust.edu.cn (X.D.)

[2] School of Mechanical Engineering, Nanjing University of Science and Technology, Nanjing 210094, China; rifath.bin.hossain@njust.edu.cn

[3] Fujian Key Laboratory of Flexible Electronics, Strait Institute of Flexible Electronics (SIFE Future
Technologies), Fujian Normal University, Fuzhou 350117, China;
ylyang@fjnu.edu.cn

* Correspondence: fengxiangqin@njust.edu.cn

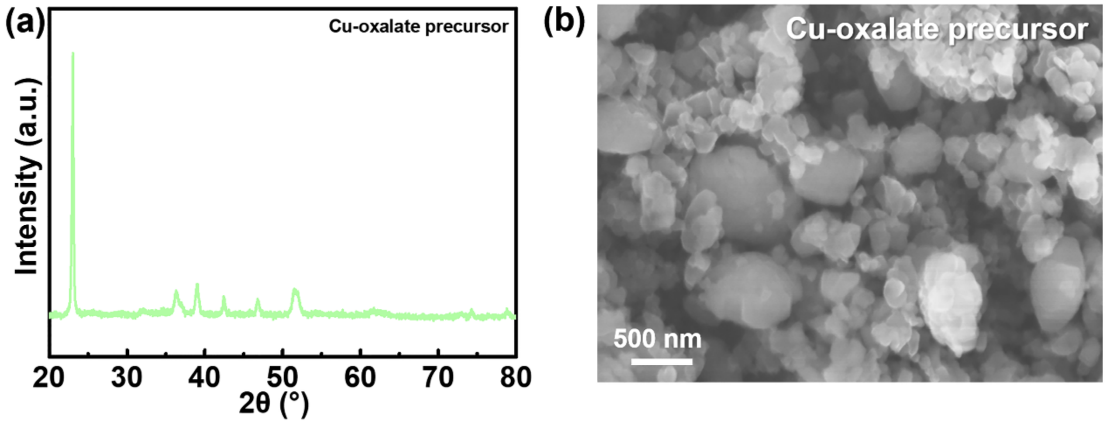

Figure S1. Characterization of the as-prepared copper oxalate precursor: (a) X-ray diffraction (XRD) pattern and (b) scanning electron microscopy (SEM) image.

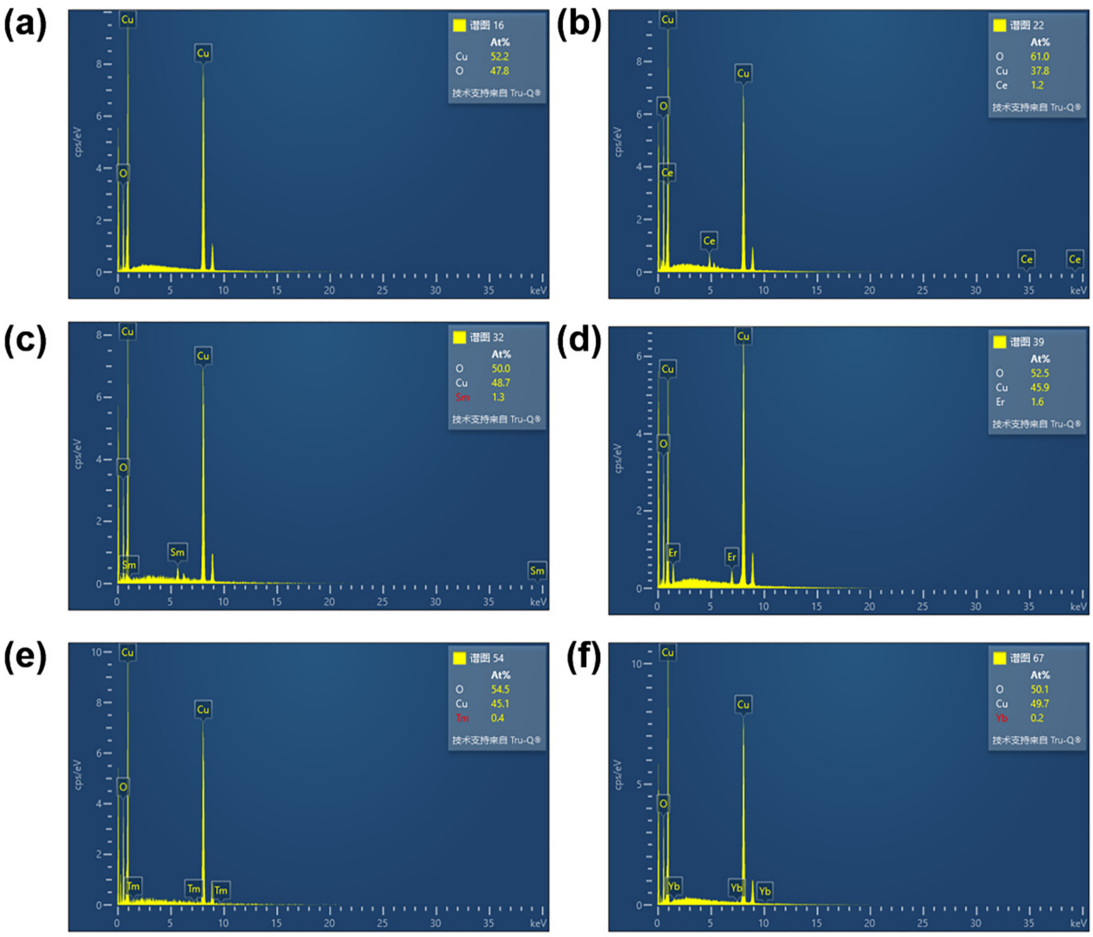

Figure S2. EDS spectra of (a) CuO, (b) Ce-CuO, (c) Sm-CuO, (d) Er-CuO, (e) Tm-CuO, and (f) Yb-CuO, showing Cu and O signals together with detectable low-level RE signals in the corresponding modified samples.

Table S1. Semi-quantitative EDS elemental compositions of pristine and RE-modified copper-oxalate-derived CuO samples.

Element / Photocatalyst	Cu	O	RE
**CuO**	52.2	47.8	—
**Ce-CuO**	37.8	61.0	1.2
**Sm-CuO**	48.7	50.0	1.3
**Er-CuO**	45.9	52.5	1.6
**Tm-CuO**	45.1	54.5	0.4
**Yb-CuO**	49.7	50.1	0.2

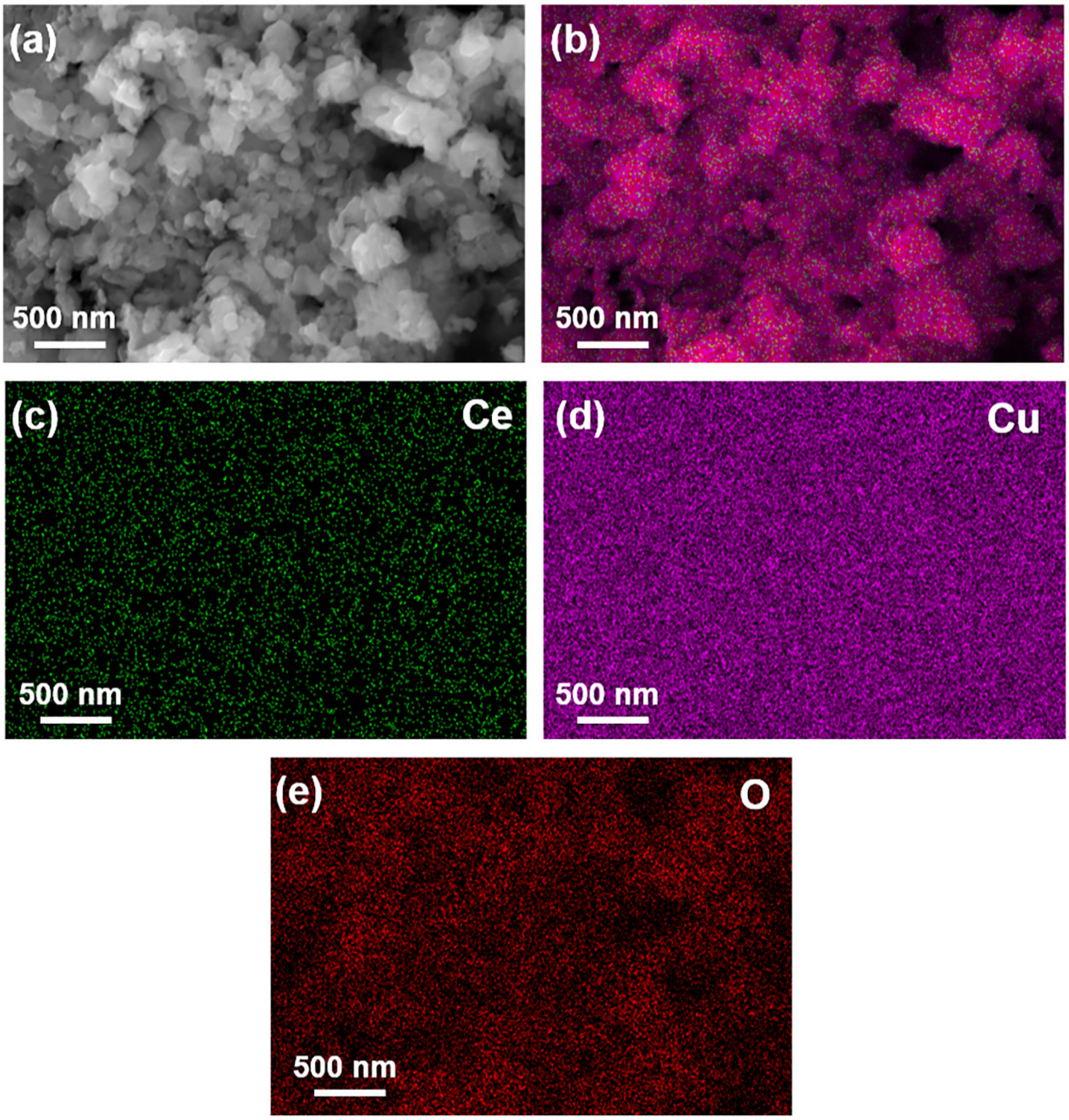

Figure S3. EDS elemental mapping of as-prepared Ce-CuO: (a) SEM image, (b) merged elemental map, and individual elemental distributions of (c) Ce, (d) Cu, and (e) O.

Table S2. Diffraction peak positions, interplanar spacings, and apparent lattice-perturbation magnitudes of pristine and RE-modified copper-oxalate-derived CuO calculated from the (−111) and (111) planes.

Photocatalyst	2θ (°)	d(-111) (nm)	Perturbation (-111) (%)	2θ (°)	d(111) (nm)	Perturbation (111) (%)
**CuO**	35.78	0.2506	—	38.99	0.2307	—
**Ce-CuO**	35.89	0.2498	−0.32	39.05	0.2304	−0.13
**Sm-CuO**	35.87	0.2500	−0.24	39.03	0.2305	−0.09
**Er-CuO**	35.85	0.2501	−0.20	39.04	0.2304	−0.13
**Tm-CuO**	35.68	0.2512	+0.24	38.89	0.2311	+0.17
**Yb-CuO**	35.78	0.2506	0.00	38.98	0.2307	0.00

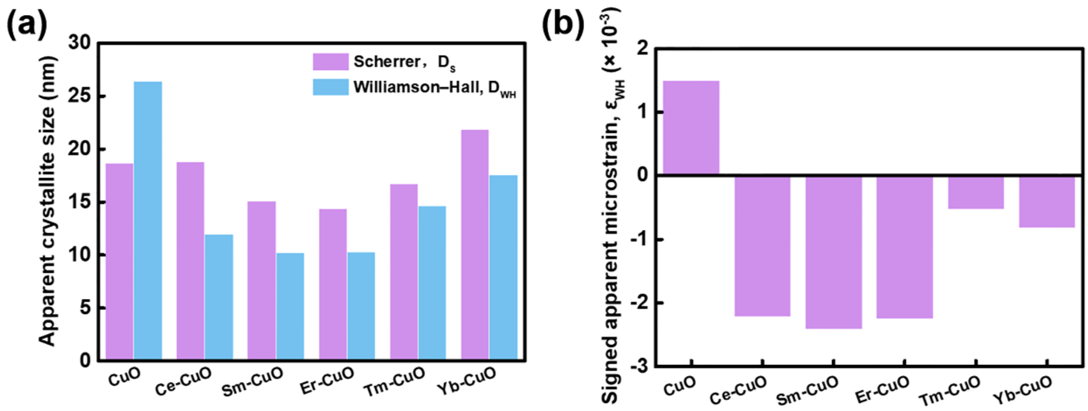

Figure S4. XRD peak-broadening-derived structural parameters of pristine and RE-modified CuO: (a) comparison of the apparent Scherrer crystallite size D_S and Williamson–Hall crystallite size D_{WH}, and (b) signed apparent Williamson–Hall microstrain ε_{WH}.

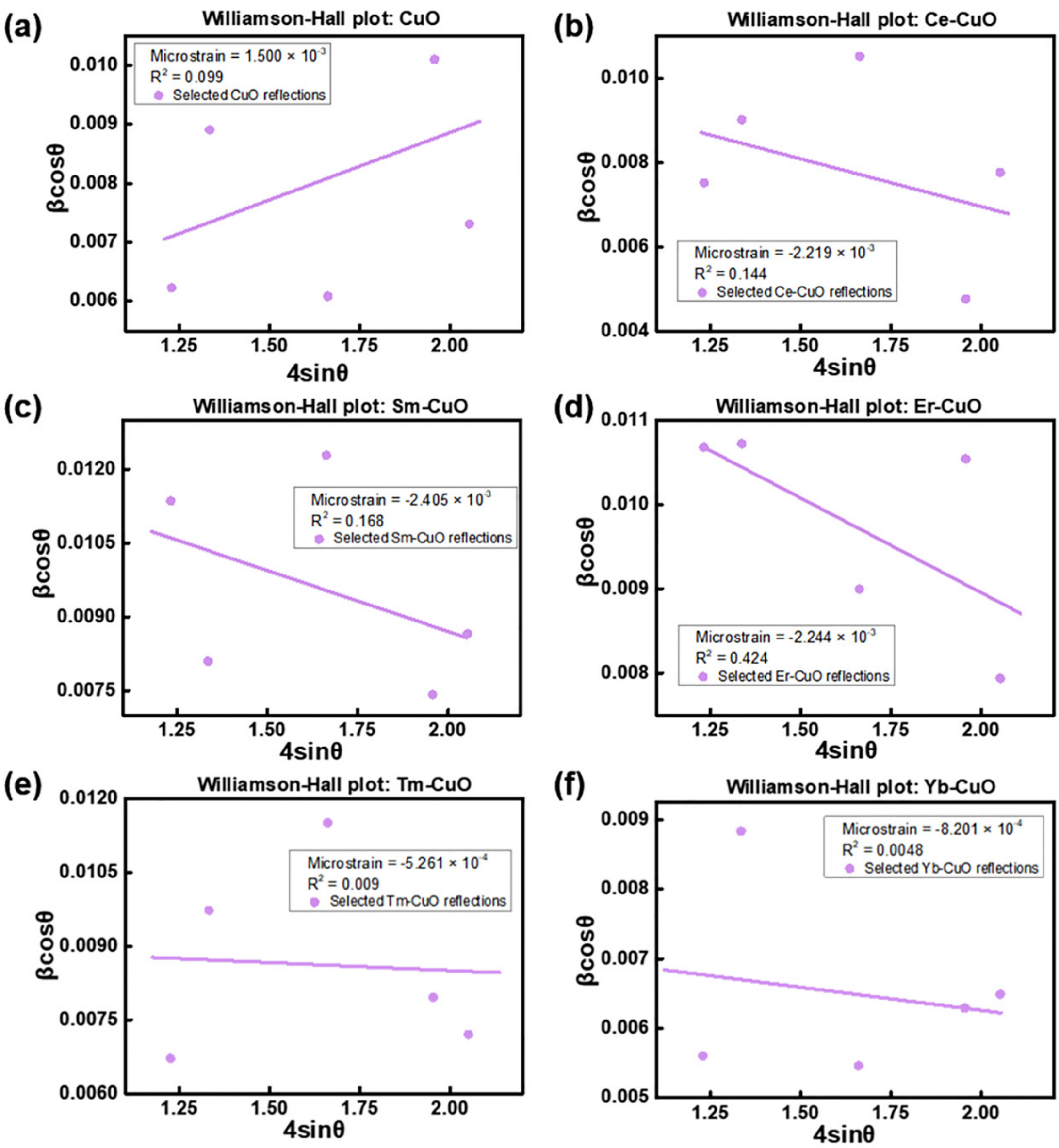

Figure S5. Williamson–Hall plots of (a) CuO, (b) Ce-CuO, (c) Sm-CuO, (d) Er-CuO, (e) Tm-CuO, and (f) Yb-CuO based on five selected monoclinic CuO reflections. Because no instrumental-broadening correction was applied and the fitting linearity was limited for several samples, the resulting parameters are treated as comparative apparent estimates.

Table S3. Apparent crystallite sizes and signed Williamson–Hall microstrain parameters of pristine and RE-modified CuO.

Sample	D_S (nm)	SD of D_S (nm)	D_{WH} (nm)	ε_{WH} (×10^{-3})	W–H (R^2)
CuO	18.66	4.00	26.38	+1.500	0.099
Ce-CuO	18.82	6.15	11.99	−2.219	0.144
Sm-CuO	15.07	3.19	10.25	−2.405	0.168
Er-CuO	14.39	2.01	10.29	−2.244	0.424
Tm-CuO	16.73	3.55	14.62	−0.526	0.009
Yb-CuO	21.85	3.84	17.58	−0.820	0.048

Note: D_S represents the mean apparent crystallite size calculated using the Scherrer equation from the (−111) ,(111) ,(−202) ,(202) ,and (−113) CuO reflections. The corresponding SD represents the dispersion among the five selected reflections rather than experimental repeatability. D_{WH} and $\varepsilon_{\mathrm{WH}}$ were obtained from the intercept and slope of the Williamson–Hall linear fit, respectively. No instrumental-broadening correction was applied; therefore, the reported crystallite-size and microstrain values are treated as apparent estimates.

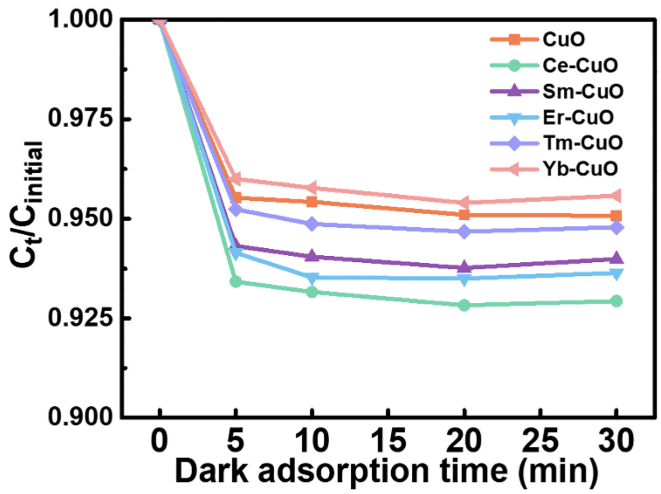

Figure S6. Dark-adsorption profiles of methyl orange over pristine and RE-modified CuO during 30 min of dark stirring, expressed as $C_t/C_{initial}$.

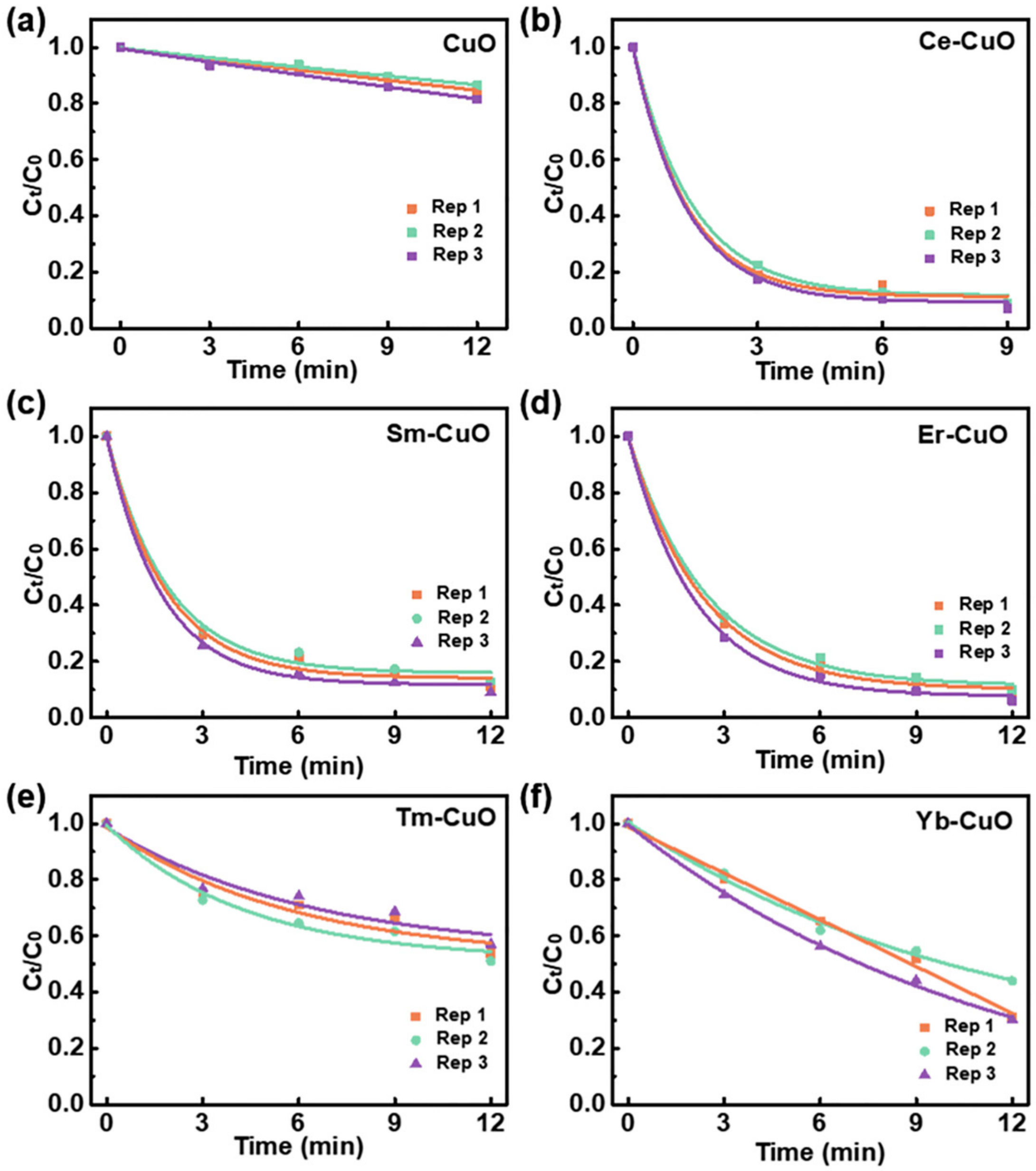

Figure S7. Results of three independent photocatalytic decolorization experiments for (a) CuO, (b) Ce-CuO, (c) Sm-CuO, (d) Er-CuO, (e) Tm-CuO, and (f) Yb-CuO under otherwise identical simulated-solar-irradiation conditions.

Table S4. Replicate decolorization efficiencies and apparent pseudo-first-order kinetic parameters.

Sample	Assessment time (min)	Rep 1 (%)	Rep 2 (%)	Rep 3 (%)	Mean ± SD (%)	k_1 (min^{-1})	k_2 (min^{-1})	k_3 (min^{-1})	Mean k_{obs} ± SD (min^{-1})	(R^2) range
**CuO**	12	15.38	13.50	18.50	15.79 ± 2.53	0.0131	0.0120	0.0170	0.0140 ± 0.0026	0.9711–0.9838
**Ce-CuO**	9	91.59	91.29	93.10	91.99 ± 0.97	0.3104	0.2927	0.3406	0.3146 ± 0.0242	0.8131–0.8674
**Sm-CuO**	12	89.23	87.02	91.00	89.08 ± 2.00	0.2154	0.1943	0.2320	0.2139 ± 0.0189	0.7765–0.8098
**Er-CuO**	12	91.33	90.00	94.01	91.78 ± 2.04	0.2342	0.2125	0.2597	0.2355 ± 0.0236	0.9027–0.9114
**Tm-CuO**	12	46.39	49.10	43.13	46.20 ± 3.01	0.0496	0.0593	0.0472	0.0520 ± 0.0064	0.8799–0.8936
**Yb-CuO**	12	68.86	56.03	70.00	64.95 ± 7.77	0.0753	0.0694	0.0969	0.0805 ± 0.0145	0.9123–0.9947

Note: Values are presented as the mean ± standard deviation of three independent photocatalytic experiments (n = 3). The apparent pseudo-first-order rate constant was determined separately for each independent experiment from the linear relationship between $\ln(C_0/C_t)$ and irradiation time. Ce-CuO was evaluated up to 9 min because of its rapid decolorization, whereas the remaining catalysts were evaluated up to 12 min. The R^2 ranges correspond to the individual kinetic analyses of the three independent experiments.

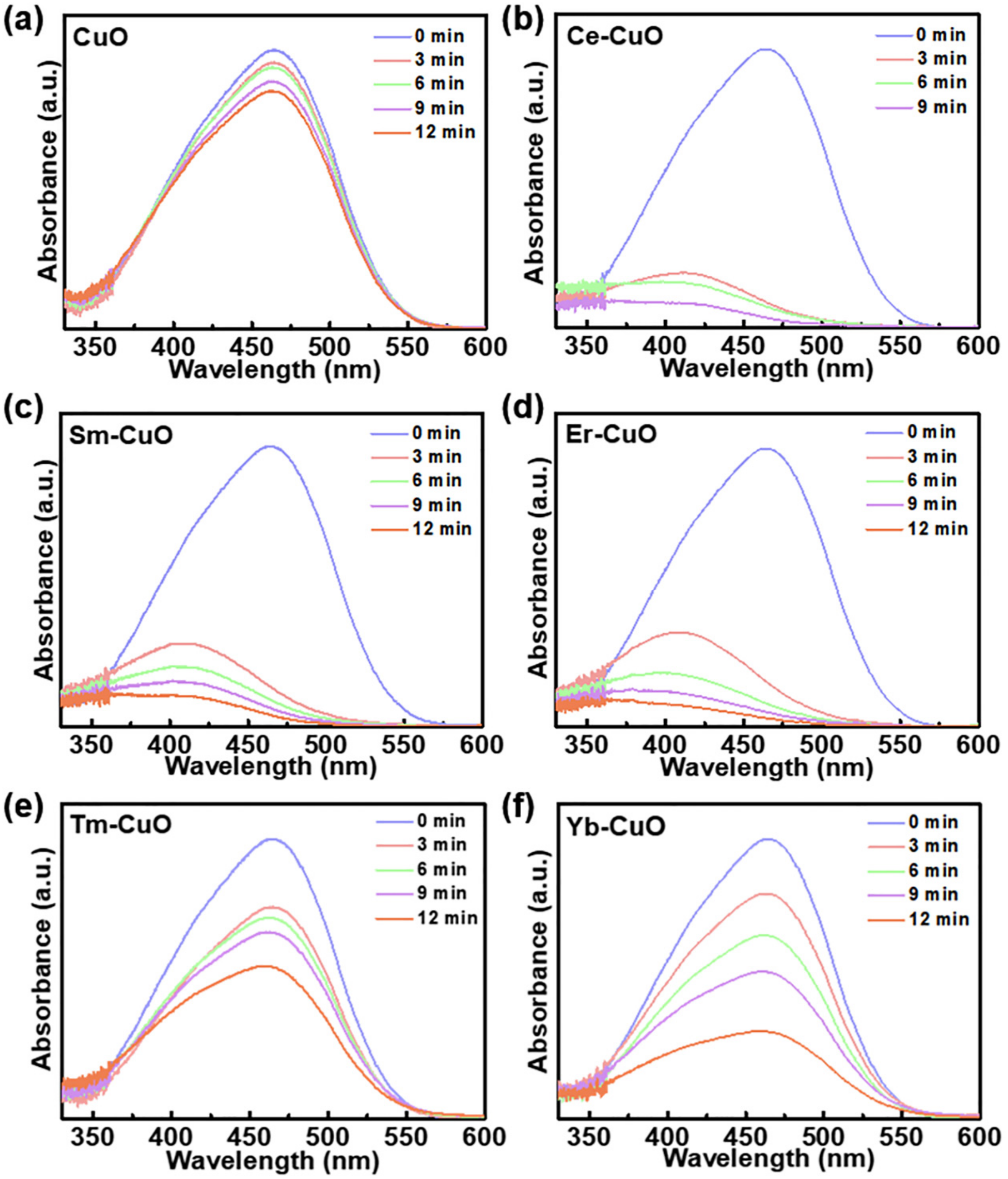

Figure S8. Time-dependent UV–vis absorption spectra of methyl orange solution during photocatalytic treatment over (a) CuO, (b) Ce-CuO, (c) Sm-CuO, (d) Er-CuO, (e) Tm-CuO, and (f) Yb-CuO under simulated solar irradiation.

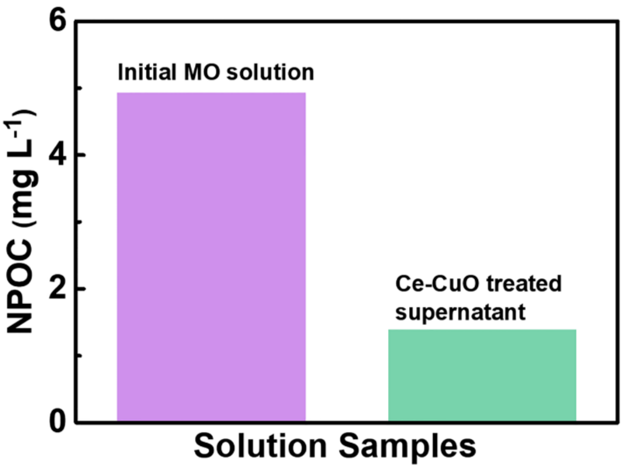

Figure S9. NPOC concentrations of the initial MO solution and the Ce-CuO-treated supernatant after simulated-solar photocatalytic treatment.

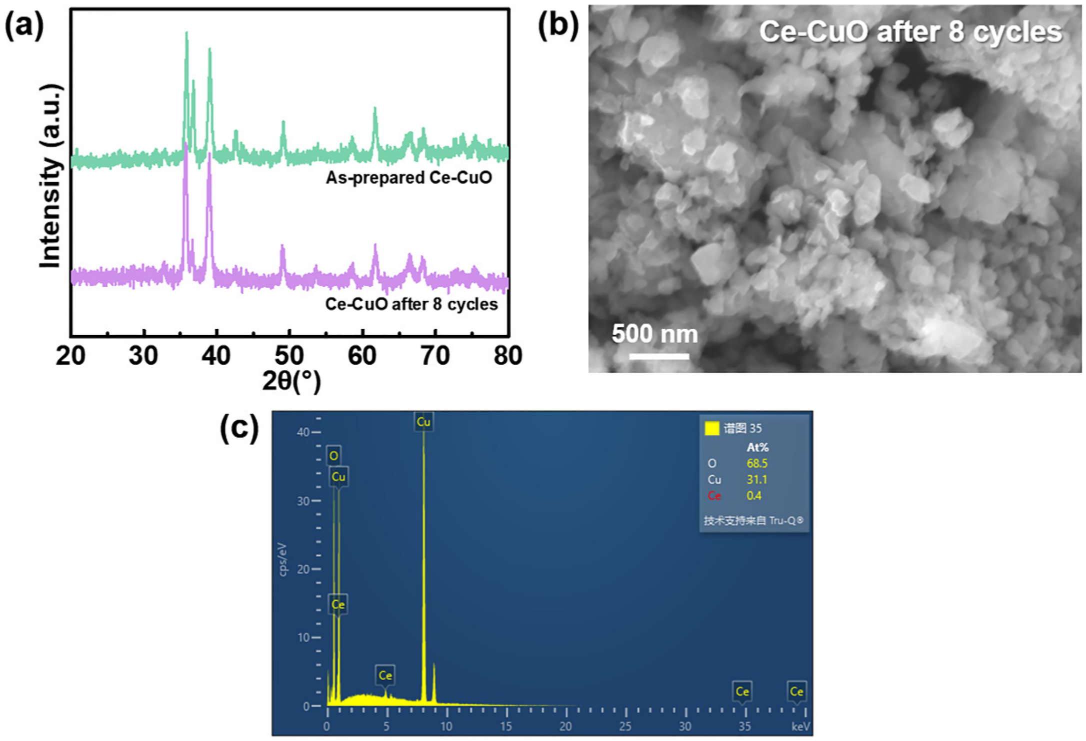

Figure S10. Post-cycling characterization of Ce-CuO recovered after eight consecutive photocatalytic cycles: (a) comparison of the XRD patterns of as-prepared and cycled Ce-CuO, (b) SEM image of the recovered catalyst, and (c) corresponding EDS spectrum.

Table S5. Local semi-quantitative EDS elemental compositions of as-prepared Ce-CuO and Ce-CuO recovered after eight photocatalytic cycles.

Sample	Cu (at.%)	O (at.%)	Ce (at.%)
**As-prepared Ce-CuO**	37.8	61.0	1.2
**Ce-CuO after 8 cycles**	31.1	68.5	0.4

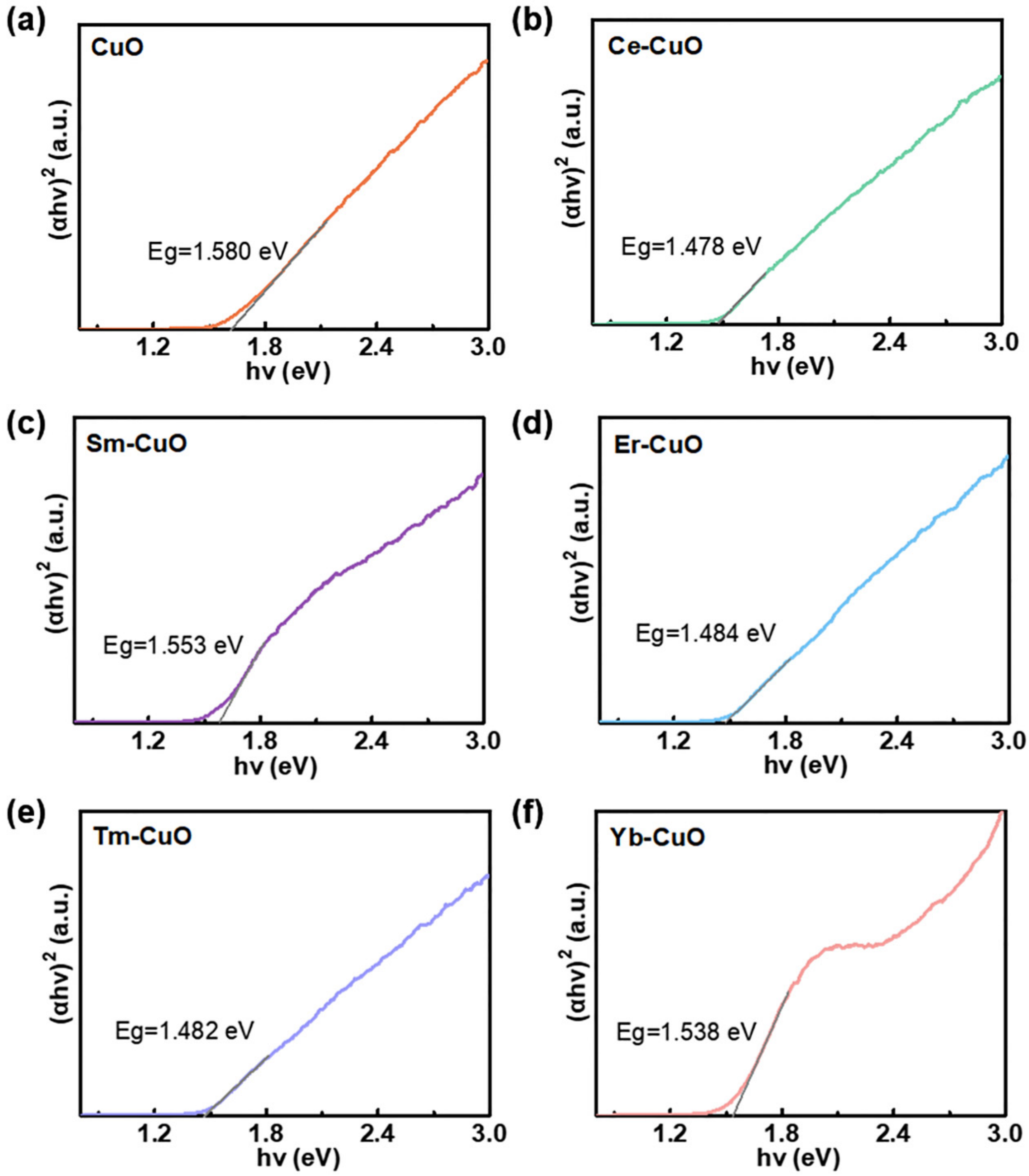

Figure S11. Complete direct-allowed Tauc plots of (a) CuO, (b) Ce-CuO, (c) Sm-CuO, (d) Er-CuO, (e) Tm-CuO, and (f) Yb-CuO.

Table S6. Fitting-range sensitivity of the Mott–Schottky-derived apparent majority-carrier-density descriptors.

Sample	$N_{A,1}$ (cm^{-3})	$N_{A,2}$ (cm^{-3})	$N_{A,3}$ (cm^{-3})	Mean N_A (cm^{-3})	Fitting-range SD (cm^{-3})	RSD (%)	R^2 range
**CuO**	5.60×10^{19}	5.99×10^{19}	6.02×10^{19}	5.87×10^{19}	0.23×10^{19}	3.97	0.9821 – 0.9954
**Ce-CuO**	1.90×10^{21}	1.97×10^{21}	1.96×10^{21}	1.94×10^{21}	0.04×10^{21}	1.99	0.9928 – 0.9967
**Sm-CuO**	1.24×10^{21}	1.47×10^{21}	1.48×10^{21}	1.40×10^{21}	0.14×10^{21}	9.79	0.9254 – 0.9828
**Er-CuO**	3.12×10^{20}	3.30×10^{20}	3.25×10^{20}	3.22×10^{20}	0.09×10^{20}	2.92	0.9802 – 0.9890
**Tm-CuO**	1.38×10^{20}	1.34×10^{20}	1.36×10^{20}	1.36×10^{20}	0.02×10^{20}	1.64	0.9856 – 0.9903
**Yb-CuO**	1.48×10^{20}	1.56×10^{20}	1.55×10^{20}	1.53×10^{20}	0.04×10^{20}	2.83	0.9887 – 0.9965

Note: $N_{A,1}$, $N_{A,2}$, and $N_{A,3}$ were obtained by fitting three adjacent negative-slope linear regions selected from the same Mott–Schottky curve. The same geometric electrode area (0.0616 cm^2), relative dielectric constant (ε_r = 12), and carrier-density calculation procedure were applied in all three analyses. The dielectric constant was adopted as a common approximation for internally consistent comparison and was not independently measured for the individual RE-modified samples. The reported mean, fitting-range standard deviation, and relative standard deviation represent fitting-range sensitivity rather than experimental repeatability. The R^2 range represents the minimum and maximum coefficients of determination obtained from the three linear fits. The three adjacent fitting windows used in Table S6 were selected specifically to evaluate the sensitivity of the calculated apparent N_A descriptor to fitting-range selection. These fitting windows are distinct from the separate representative negative-slope linear fit used to determine the x-axis intercept and apparent E_{fb} values reported in Table S7.

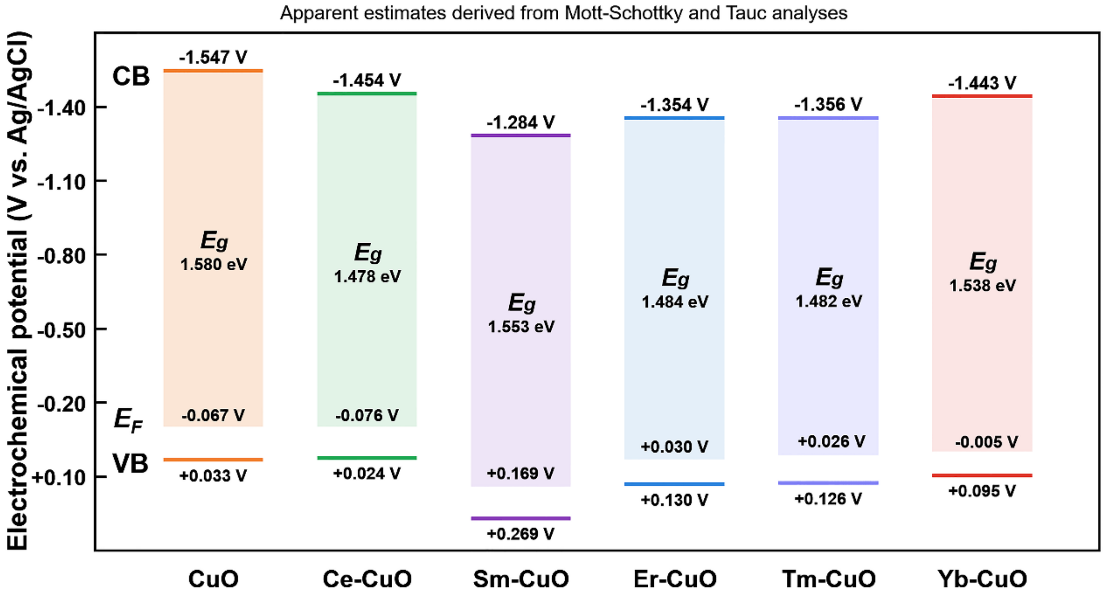

Figure S12. Comparative apparent conduction-band, valence-band, and Fermi-level positions of pristine and RE-modified CuO estimated by combining Mott–Schottky and Tauc analyses. All potentials are reported versus Ag/AgCl. The displayed values are comparative apparent estimates rather than direct absolute energy-level measurements.

Table S7. Apparent optical band-gap, flat-band/Fermi-level, valence-band, and conduction-band positions of pristine and RE-modified CuO estimated from Mott–Schottky and Tauc analyses.

Sample	Apparent E_g (eV)	Apparent $E_{fb} \approx E_F$ (V vs. Ag/AgCl)	Apparent E_{VB} (V vs. Ag/AgCl)	Apparent E_{CB} (V vs. Ag/AgCl)	M–S R^2
CuO	1.580	−0.067	+0.033	−1.547	0.953
Ce-CuO	1.478	−0.076	+0.024	−1.454	0.955
Sm-CuO	1.553	+0.169	+0.269	−1.284	0.983
Er-CuO	1.484	+0.030	+0.130	−1.354	0.962
Tm-CuO	1.482	+0.026	+0.126	−1.356	0.953
Yb-CuO	1.538	−0.005	+0.095	−1.443	0.953

Note: The apparent E_{fb} values were calculated from the x-axis intercepts of separately selected representative negative-slope Mott–Schottky fits after inclusion of the thermal term at 298 K. The corresponding M–S R^2 values reported in this table refer only to these representative fits used for intercept and band-edge estimation. They are not the R^2 values of the three adjacent fitting windows used for the apparent N_A fitting-range-sensitivity analysis in Table S6. Consequently, the R^2 values in Tables S6 and S7 describe two different fitting procedures with different analytical purposes and are not expected to be identical or to fall within the same numerical range. The apparent Fermi-level position was approximated as $E_F \approx E_{fb}$; E_{VB} was estimated as E_{fb} + 0.10 V for the p-type samples; and E_{CB} was calculated from $E_{VB} - E_g$. All potentials are reported versus Ag/AgCl. These values are comparative apparent estimates and should not be interpreted as direct UPS, valence-band XPS, Kelvin-probe, or work-function measurements.

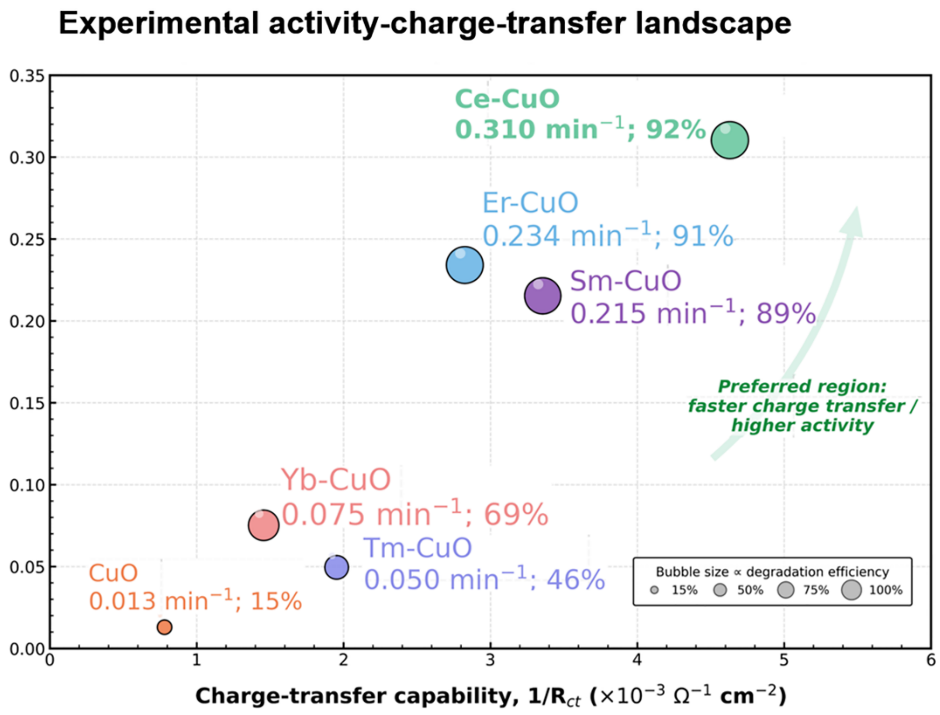

Figure S13. Descriptive relationship between the representative apparent pseudo-first-order rate constant k_{obs} and inverse fitted charge-transfer resistance $1/R_{ct}$ for pristine and RE-modified CuO. Bubble size represents the corresponding decolorization efficiency. The plot is used only to visualize the experimental descriptors and does not imply a single-parameter causal relationship between R_{ct} and photocatalytic activity.

Table S8. Summary of the curated external photocatalytic kinetic dataset and descriptive benchmarking metrics.

Parameter	Value	Interpretation
Curated external dataset size	439 entries	Deduplicated kinetic reference space
Number of literature references	54	Source-level grouped evaluation used
Full external median k	0.0130 min^{-1}	Central kinetic reference
Full external IQR	0.0033–0.0275 min^{-1}	Typical kinetic range
Full external 95th percentile	0.0751 min^{-1}	High-activity threshold in the curated dataset
Curated external maximum k	0.1000 min^{-1}	Maximum within the curated dataset, not full literature maximum
Visible-light subset size	44 entries	Visible-light comparison group
Visible-light median k	0.0094 min^{-1}	Visible-light kinetic reference
CuO k_{obs}	0.0131 min^{-1}	Close to external median
Ce-CuO k_{obs}	0.3104 min^{-1}	Highest among prepared samples
Ce-CuO vs full external median	23.9×	Strong external kinetic enhancement
Ce-CuO vs visible-light median	33.0×	Strong visible-light contextual advantage
Best repeated grouped model performance	RMSE = 0.5587; R^2 = −0.0033	Weak predictive robustness; contextualization only

Note: The external literature records were obtained under heterogeneous experimental conditions. The summary provides descriptive kinetic context and is not interpreted as a condition-normalized performance ranking.

Table S9. Construction and preprocessing of the curated external photocatalytic kinetic dataset used for literature-derived machine-learning-assisted contextualization.

Item	Description
Raw dataset size	449 entries
Final dataset size	439 entries after exact duplicate removal
Number of references	54 literature references
Target variable	Apparent photocatalytic rate constant, k, standardized as min^{-1}
Transformed target	$\log_{10}(k)$
Photocatalyst categories	6
Contaminant categories	47
Irradiation categories	3
Input descriptors	Photocatalyst identity, contaminant identity, irradiation type, dosage, particle-size descriptor, initial concentration, pH
Missing-value treatment	Numerical imputation; missingness indicators for incomplete literature-reported descriptors
Splitting strategy	Reference-grouped splitting
Purpose	Literature-derived machine-learning-assisted kinetic contextualization only

Table S10. Repeated reference-grouped machine-learning model robustness.

Model	Median RMSE	Median MAE	Median R^2	Interpretation
RandomForest	0.5587	0.4497	−0.0033	Best by median RMSE, but weak predictive robustness
GradientBoosting	0.5760	0.4670	−0.1002	Not robust
HistGradientBoosting	0.5785	0.4491	−0.1966	Not robust
DummyMedian	0.6029	—	−0.1347	Baseline
ElasticNet	0.5951	0.5043	−0.2013	Weak
Ridge	0.6260	—	−0.3565	Weak

Note: Because repeated reference-grouped evaluation showed weak predictive robustness, the machine-learning model outputs were used only to define the claim boundary and support literature-derived machine-learning-assisted kinetic contextualization, not catalyst discovery, direct prediction, or mechanistic proof.

Table S11. Detailed machine-learning-assisted kinetic contextualization of CuO and RE-CuO samples.

Sample	k / min^{-1}	Relative to external median	Relative to visible-light median	Position in curated external dataset
CuO	0.0131	1.0×	1.4×	Near external median
Tm-CuO	0.0496	3.8×	5.3×	Above median
Yb-CuO	0.0753	5.8×	8.0×	Near 95th percentile
Sm-CuO	0.2154	16.6×	22.9×	Above curated external maximum
Er-CuO	0.2342	18.0×	24.9×	Above curated external maximum
Ce-CuO	0.3104	23.9×	33.0×	Above curated external maximum

Note: The curated external maximum refers only to the maximum value within the external dataset used in this work and should not be interpreted as the maximum value across the entire photocatalysis literature. The external visible-light subset was included as an auxiliary visible-region-related reference because the visible region represents a major photoactive portion of the simulated solar spectrum for narrow-bandgap CuO-based photocatalysts; however, it should not be regarded as a strictly condition-matched benchmark for the present simulated-solar experiments.

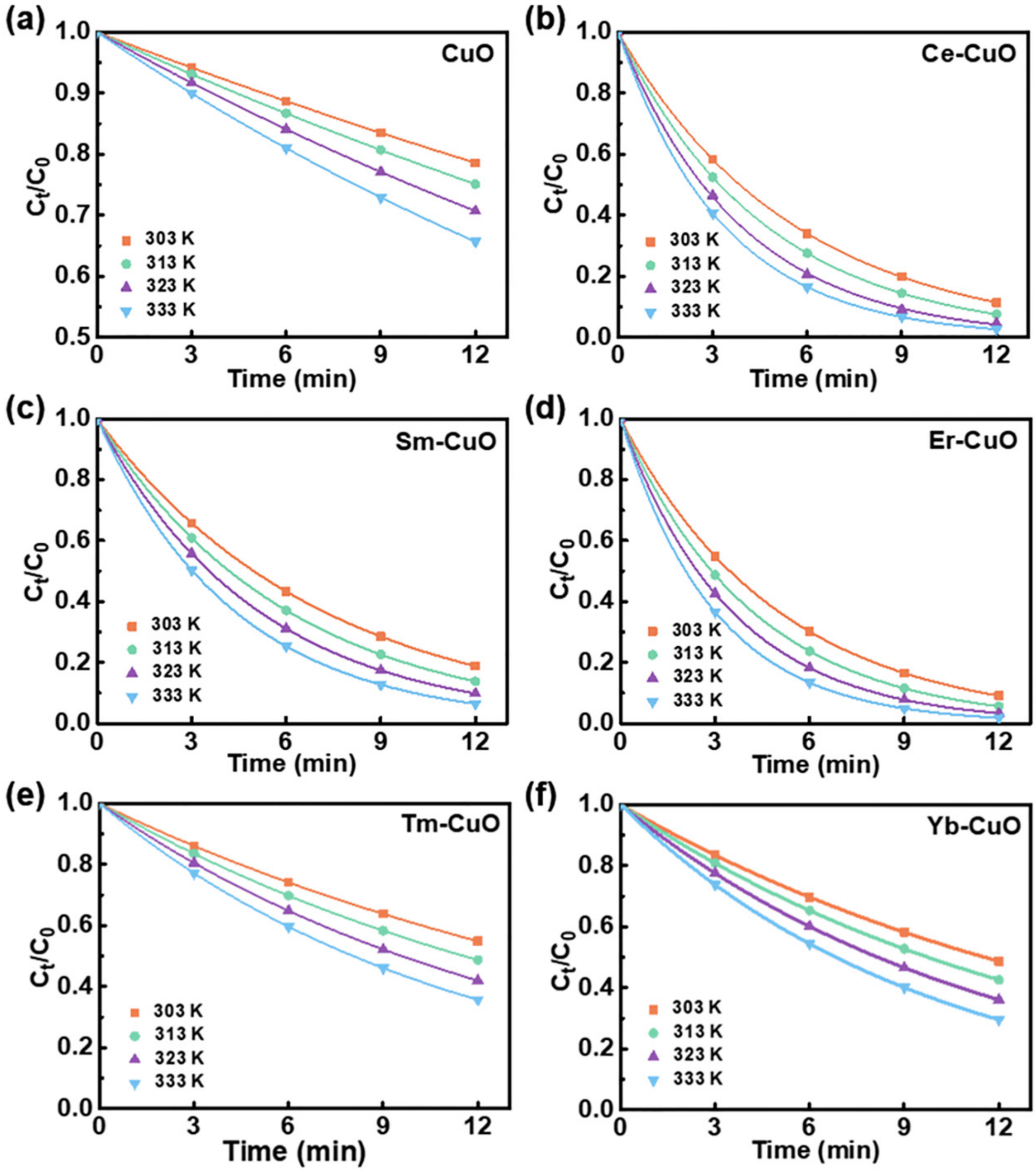

Figure S14. Temperature-dependent photocatalytic decolorization profiles of methyl orange (MO) over (a) CuO, (b) Ce-CuO, (c) Sm-CuO, (d) Er-CuO, (e) Tm-CuO, and (f) Yb-CuO at 303–333 K under simulated solar irradiation, expressed as C_t/C_0 versus reaction time.
